# Arylaminopropanone Derivatives as Potential Cholinesterase Inhibitors: Synthesis, Docking Study and Biological Evaluation

**DOI:** 10.3390/molecules25071751

**Published:** 2020-04-10

**Authors:** Anna Hudcová, Aleš Kroutil, Renata Kubínová, Adriana D. Garro, Lucas J. Gutierrez, Daniel Enriz, Michal Oravec, Jozef Csöllei

**Affiliations:** 1 Department of Chemical Drugs, Faculty of Pharmacy, University of Veterinary and Pharmaceutical Sciences, Palackeho tr. 1, 61242 Brno, Czech Republic; hudcova.anna@seznam.cz (A.H.); csolleij@vfu.cz (J.C.); 2 Department of Natural Drugs, Faculty of Pharmacy, University of Veterinary and Pharmaceutical Sciences, Palackeho tr. 1, 61242 Brno, Czech Republic; kubinovar@vfu.cz; 3 Facultad de Química, Bioquímica y Farmacia, Universidad Nacional de San Luis, Chacabuco 915, San Luis 5700, Argentina; adrianagarrosl@gmail.com (A.D.G.); lucasg@unsl.edu.ar (L.J.G.); danielenriz@gmail.com (D.E.); 4 IMIBIO-CONICET, UNSL, Chacabuco 915, San Luis 5700, Argentina; 5 Global Change Research Institute CAS, Belidla 986/4a, 60300 Brno, Czech Republic; oravec.m@czechglobe.cz

**Keywords:** arylaminopropanone, *N*-phenylcarbamate, acetylcholinesterase, butyrylcholinesterase, enzyme assays, molecular modelling

## Abstract

Neurodegenerative diseases in which the decrease of the acetylcholine is observed are growing worldwide. In the present study, a series of new arylaminopropanone derivatives with *N*-phenylcarbamate moiety (**1–16**) were prepared as potential acetylcholinesterase and butyrylcholinesterase inhibitors. In vitro enzyme assays were performed; the results are expressed as a percentage of inhibition and the IC_50_ values. The inhibitory activities were compared with reference drugs galantamine and rivastigmine showing piperidine derivatives (**1–3**) as the most potent. A possible mechanism of action for these compounds was determined from a molecular modelling study by using combined techniques of docking, molecular dynamics simulations and quantum mechanics calculations.

## 1. Introduction

Dementia is a syndrome caused by a variety of brain illnesses, whose worldwide incidence and prevalence is still growing. Alzheimer’s disease (AD) is the most common form of dementia. It includes more than 65 % diagnostic cases representing 5.4 million new patients every year. This neurodegenerative disease is characterized by the disturbance of multiple higher cortical functions like memory, orientation, comprehension, and other cognitive functions [1]. AD is progressive, leading to an irreversible loss of neurons. According to the cholinergic hypothesis, a loss of cholinergic activity is commonly observed in the brains of AD patients and experimental data confirmed the role of acetylcholine (ACh) in learning and memory [2,3].

Acetylcholinesterase (AChE, EC 3.1.1.7) and butyrylcholinesterase (BuChE, EC 3.1.1.8) are the enzymes that play a role in cholinergic transmission [4]. Both are expressed in all types of tissues, but the activity varies. AChE can be found mainly in the brain, muscles and cholinergic neurons and it is responsible for rapid hydrolysis of acetylcholine on synapses [5]. BuChE is expressed in neurons, glia cells and endothelial cells, and it is present in serum, liver, kidney or the intestine. The physiological role of BuChE in acetylcholine hydrolysis is minor. Its importance grows in the late stage of AD when the level of AChE declines [4,6].

AChE occurs in a number of molecular forms, differing in both quaternary structure and the mode of anchoring within the synapse [5]. The expression pattern varies between species, even from tissue to tissue of the same organism. The solution of the three-dimensional structure of *Torpedo californica* AChE (TcAChE) [7] enhanced the understanding of the structural elements underlying its specificity and catalytic power. Succeeding elucidation of the human [8] and *Drosophila* [9] AChE structures permitted a structure-based approach to the design of anticholinesterase (antiChE) drugs and insecticides. Nowadays, there are more than 250 AChE structures solved for different species deposited in the Protein Data Bank [10]. The crystal structure of human BuChE is known for both native and recombinant variants [11].

Different kinetic, crystallographic or calorimetric studies indicate that the active site of both cholinesterases (ChEs) contains two subsites, the ‘esteratic’ and ‘anionic’ subsites, corresponding to the catalytic machinery and the choline-binding pocket [12,13]. The catalytic triad that forms the active site consists of Ser, Glu and His [11,14,15].

In the treatment of AD, one class of compounds is acting via the inhibition of AChE and/or BuChE, increasing the level of ACh on synapses. Donepezile, galantamine and rivastigmine were the first AChE inhibitors in clinical practice and they still have their place in current therapy. The effect, activity and bonding of these compounds are well known, they are used both as standards and as initial structures in research [16,17,18].

The important class of ChE inhibitors includes compounds with at least one carbamate group, like rivastigmine, physostigmine, neostigmine and their derivatives. They are classified as the pseudo-irreversible inhibitors, as the inhibition of the enzyme is time-dependent. Carbamate moiety forms a complex with the serine residue of the catalytic triad [13]. The non-carbamate part of the ligand is responsible for selectivity and binding dynamics [19].

Structures with carbamate moiety have a lot of beneficial biological and pharmacological properties, such as chemical stability, their capability to permeate cell membranes, and their participation in hydrogen bonding to amino and carboxyl groups. Carbamates can be found in many drugs and prodrugs, i.e., zafirlukast (treatment of asthma), efavirenz (antiretroviral therapy), mitomycin C (antitumor antibiotic) albendazole (anthelmintic drug), or the above-mentioned rivastigmine [20]. In recent studies, several carbamate compounds with different selectivity on ChEs were described [21,22,23]. Also the incorporation of more carbamate moieties seems to be a very good perspective approach to the drug synthesis [24].

The present study describes the synthesis of novel carbamate derivatives with arylaminopropanone structure designed as potential cholinesterase inhibitors. Their inhibition potency against AChE and BuChE was evaluated by in vitro and in silico experiments.

## 2. Results and Discussion

### 2.1. Chemistry

**Scheme 1:**
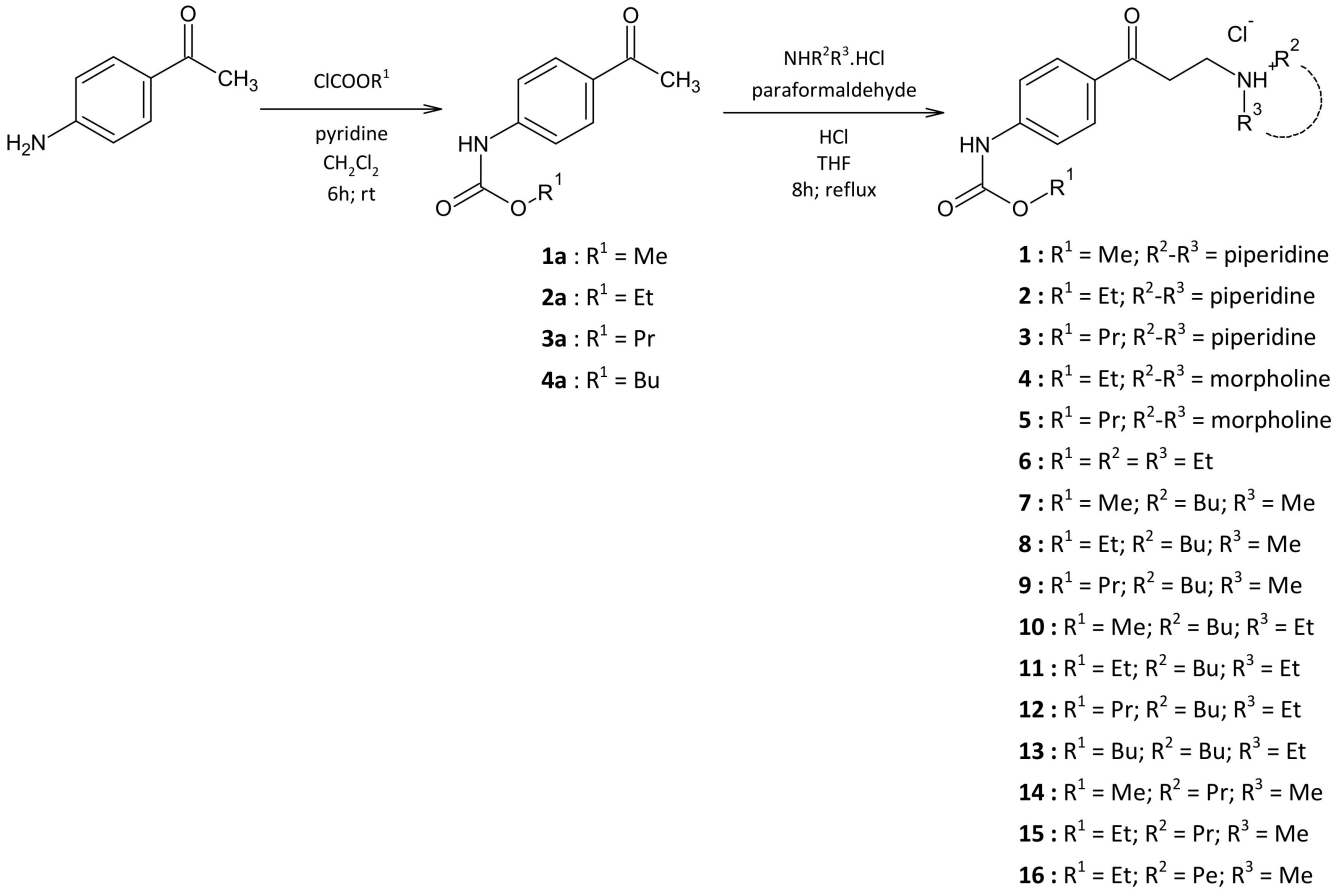
Synthesis of intermediates **1a–4a** and target compounds **1–16**, reaction conditions.

The target compounds were synthesized via two-step synthesis according to Scheme 1. The first step was the preparation of carbamates **1a–4a** by slightly modified reaction described previously [25,26]. The starting compound, 4-aminoacetophenone, was treated with alkyl-chloroformate at the presence of pyridine in dichloromethane. The replacement of acetone, originally used as a solvent, led to the improvement of the yields. The second step consisted of the Mannich reaction of carbamates **1a–4a** with paraformaldehyde and appropriate secondary amine hydrochloride. This reaction has been described using many different solvents. As a first attempt, we used propan-2-ol and ethanol, which are frequently used solvents. It resulted in poor yields and also to unacceptable purity. Significantly better results were obtained using 1,4-dioxane; finally, we changed the solvent to tetrahydrofuran. The target compounds, alkyl {4-[3-(alkyl/arylamino)-propanoyl]phenyl}carbamates (**1–16**), were isolated from the reaction mixture as hydrochlorides. The crude products were recrystallized from methanol or propan-2-ol.

### 2.2. Enzyme Assays

The in vitro testing of ChEs inhibition was performed according to the modified Ellman’s method [27]. Galantamine and rivastigmine were used as reference drugs. The percentage of inhibition activity was defined for all compounds. There were also measured the IC_50_ values for compounds with more than 50% inhibition activity. The percentage of inhibition and IC_50_ values for AChE and BuChE inhibition are given in Table 1.

According to the results, the most promising compounds are **1–3**, due to their percentage inhibition and IC_50_ values. All the compounds have a piperidine moiety in the basic part of the structure. A relatively small change in the structure—using morpholine instead of the piperidine—leads to compounds that are almost ineffective against AChE and BuChE (compounds **4** and **5**). The explanation of this phenomenon is described in *2.3.1 Docking and MD simulations* in this study.

The length of asymmetric alkyl chains in the basic part of the molecule seems to have an influence on the activity on BuChE: longer chain (butyl-, pentyl- substituents in compounds **10–13** and **16**) more active BuChE inhibitor. There can also be a visible trend of increasing activity with the prolongation of the alkyl chain in the ester part of the *N*-phenylcarbamate.

### 2.3. Molecular Modelling

In order to better understand the above-described experimental results, we conducted a study of molecular modelling by using combined techniques of simulations. This study was carried out in three different stages. A docking analysis using an Autodock program [28] was performed in the first stage. In the second stage of this study, we carried out molecular dynamics (MD) simulations using AMBER software package [29]. Using the trajectories obtained from MD simulations, an analysis per residue for the respective compounds was performed. Finally, to better appreciate the molecular interactions involved in the different L-R complexes, we performed a Quantum Theory of Atoms In Molecules (QTAIM) analysis [30] study for the most active structures of this series. The main objective of this study was to determine the possible mechanism of action of the compounds reported here and to evaluate the main molecular interactions that stabilize and destabilize the formation of the carbamates/AChE complexes. In this study, the compound used as the control, galantamine, was also included for comparison.

#### 2.3.1. Docking and MD Simulations

**Figure 1:**
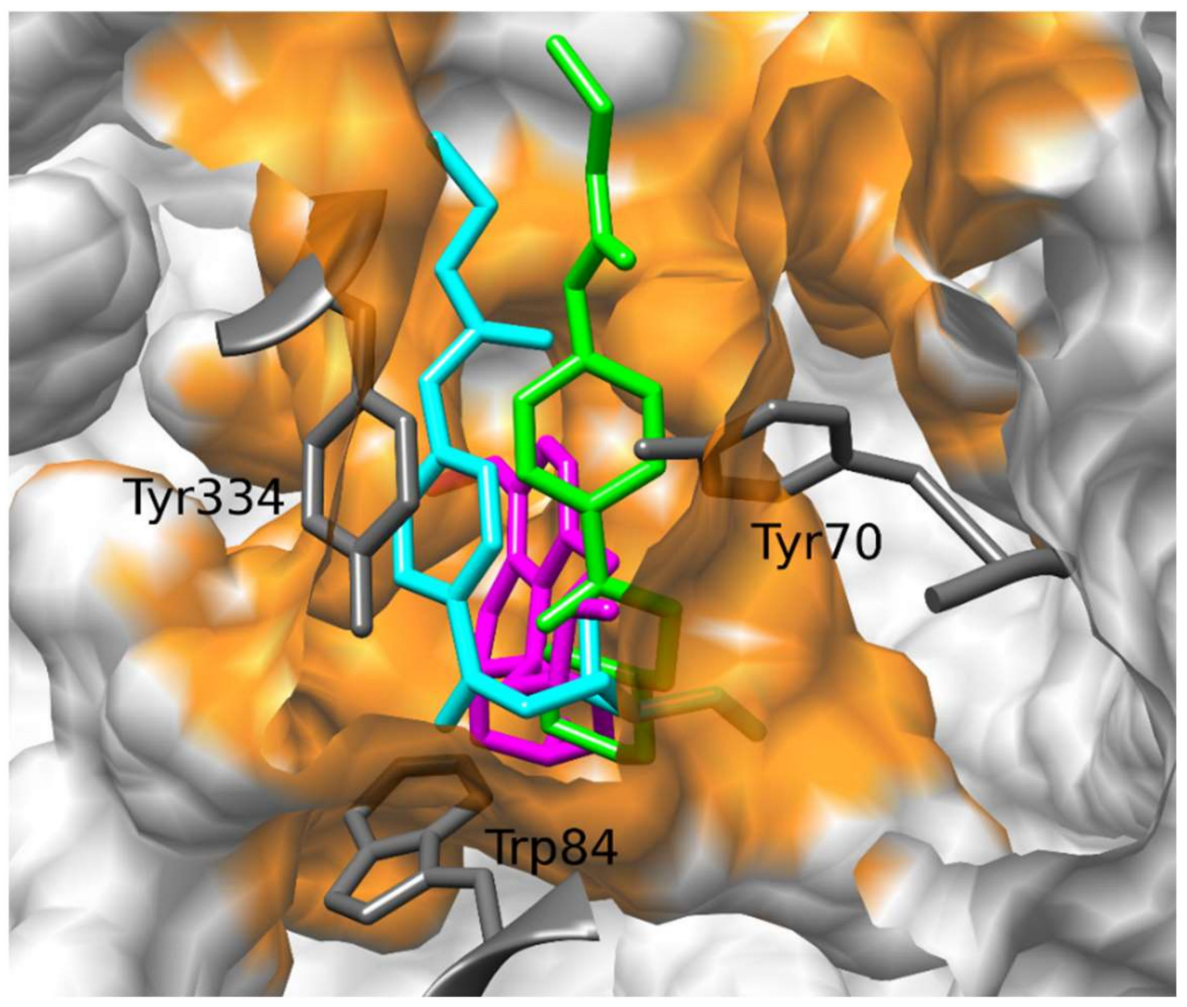
Spatial view of the compounds bonded in the binding pocket of AChE (PDB code: 1DX6). Compounds **3** (green) and **6** (light blue) are superimposed on galantamine (magenta). Some amino acids in the active site are shown in this figure for reference. The poses of compounds shown in Figure 1 were obtained from the Molecular Dynamics (MD) simulations. Residue of Ser200 cannot be observed in this figure because it is located exactly behind the position of the compounds.

The combined analysis using a docking study and MD simulations predicts that the carbamates **1–16** reported here bind in the same region of the active site of AChE to that reported for galantamine [31,32] (Figure 1); however, MD simulations indicate that these molecules are arranged spatially in a slightly different way. Therefore, these carbamates bind to some of the same amino acids of the galantamine binding site, but also to the others. In Figure 1 it can be seen that galantamine is located more deeply in the active site (binding pocket) with respect to compounds **3** and **6**. In fact these compounds are slightly displaced towards the AChE peripheral anionic site [7]. Taking as a reference the benzene ring of these ligands, compound **3** is shifted about 3.2 Å and compound **6** about 4.3 Å. Figure S3 in the Supplementary Materials shows the spatial shift among these compounds.

**Figure 2:**
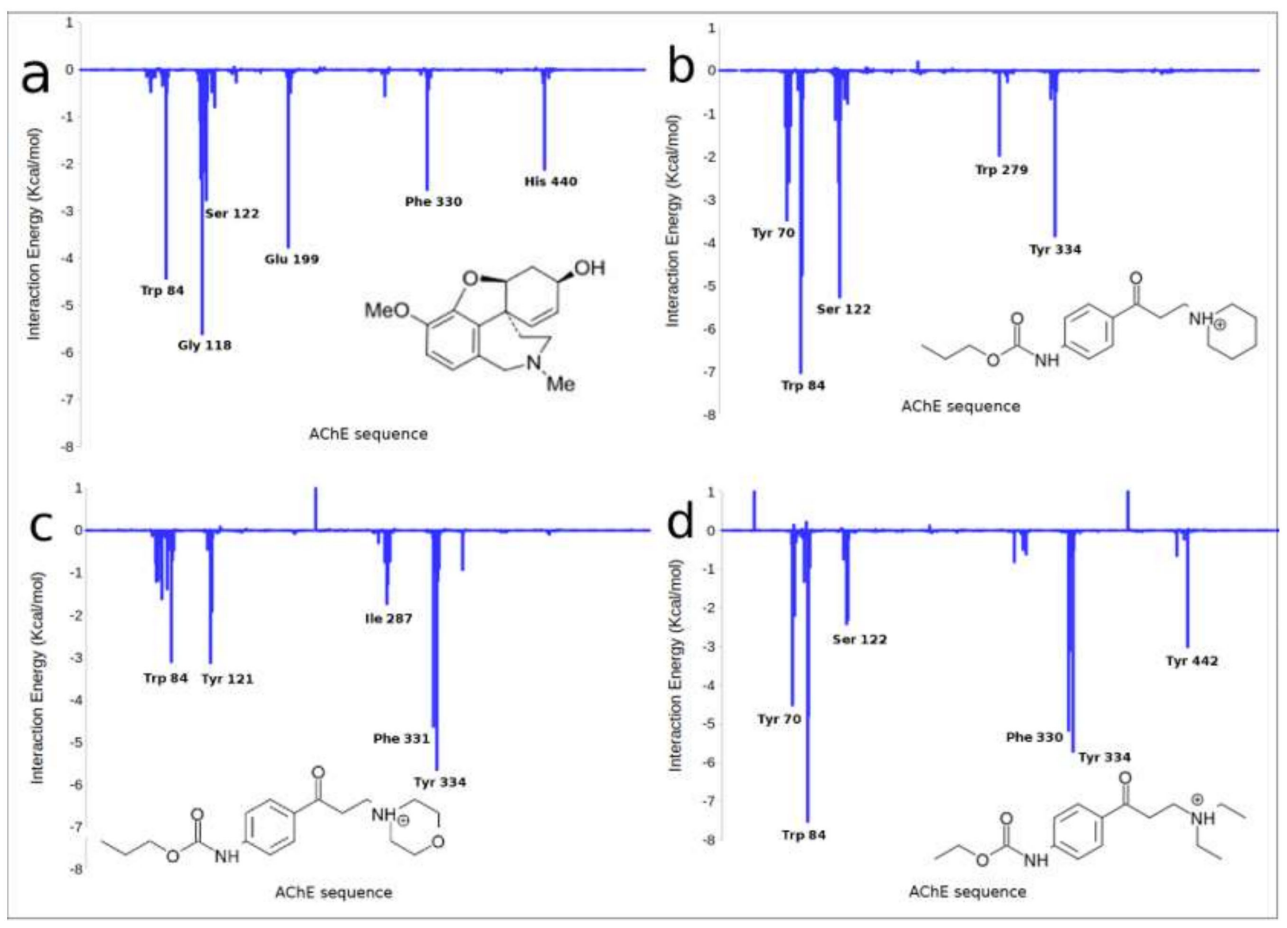
Histograms of interaction energies partitioned with respect to the AChE amino acids in complex with galantamine (**a**), compounds **3** (**b**), **5** (**c**) and **6** (**d**). The x-axis denotes the residue number of AChE, and the y-axis denotes the interaction energy between the compounds and specific residue. The negative values and positive values are favorable or unfavorable to binding, respectively.

These results can be very well appreciated in the analysis per residue which has been performed for the different compounds (Figure 2). We have previously reported that the main interactions that stabilize the galantamine complex with AChE are these with the following amino acids from the active site of the enzyme: Trp84, Gly118, Ser122, Glu199, Phe330 and His440 [32]. These interactions can be observed in the residue analysis obtained for galantamine (Figure 2a) which is very similar to that reported in the reference [32]. The histograms shown in Figure 2b–d are those obtained for compounds **3, 5** and **6**, respectively, whereas the histogram of compound **2** is reported in Supplementary Material (Figure S1). As well as for galantamine, the interactions with Trp84 and Ser122 are also important to stabilize the complexes obtained for compounds **1–3**; but other significant interactions that stabilize these complexes are those with Tyr70, Trp279 and Tyr334 (Figure 2b and Figure S1 in the Supplementary Material). Figure 2d shows the histogram obtained for compound **6**. The rest of the non-piperidine derivatives reported here (compounds **7–16**) displayed very similar binding modes to that obtained for **6**. A comparative analysis of the histograms obtained for the piperidine derivatives (compounds **1–3**; Figure 2b and Figure S1, respectively) with compound **6** (Figure 2d) allow us to observe some significant differences. It should be noted that the interaction with Ser122 is a strong interaction for compounds **1–3** (more than 5 Kcal/mol); in contrast, this interaction is very weak for the rest of the compounds reported here (less than 2 Kcal/mol) (compare Figure 2b and Figure S1 with 2d). This interaction together with other strong interactions obtained for compounds **1–3** like for example those with Tyr70, Trp279 and Tyr334 might explain, at least in part, why compounds **1-3** have greater affinity for AChE than the rest of the compounds reported here.

Particularly interesting are the results obtained for compounds **4** and **5**. It should be noted that these compounds are structurally very similar to compounds **1–3**; in fact, the only difference is the replacement of the piperidine ring with a morpholine ring. However, the combined studies of docking and MD simulations predict that these compounds are accommodated in a different spatial arrangement than that observed for compounds **1–3**. This can be observed well in the residue analysis obtained for compound **5** (Figure 2c). In this case, only the interactions with Trp84 and Tyr334 are maintained, but the important interactions with Ser122, Tyr70 and Trp279 are not present in the complex of compound **5**. It is also important to note that the interaction with Trp84 observed for compound **5** is significantly weaker than that obtained for compounds **1–3**. The different spatial ordering of compound **5** with respect to the active compounds can be observed from Figure 2c in which the main interactions are those with Tyr334 and Phe331. These results are also in agreement with the experimental data, since compounds **4** and **5** have a markedly weaker inhibitory activity in comparison with compounds **1-3**. The results obtained for compound **4** are very similar to those attained for compound **5**.

These results agree with our experimental data; however, in order to study more accurately the different interactions that stabilize and destabilize the formation of these complexes in our next stage, we conducted a QTAIM study for the complexes of the most representative compounds. In this analysis, the complex of galantamine with AChE was also evaluated, and the results are presented in the next section.

#### 2.3.2. QTAIM Analysis

**Figure 3:**
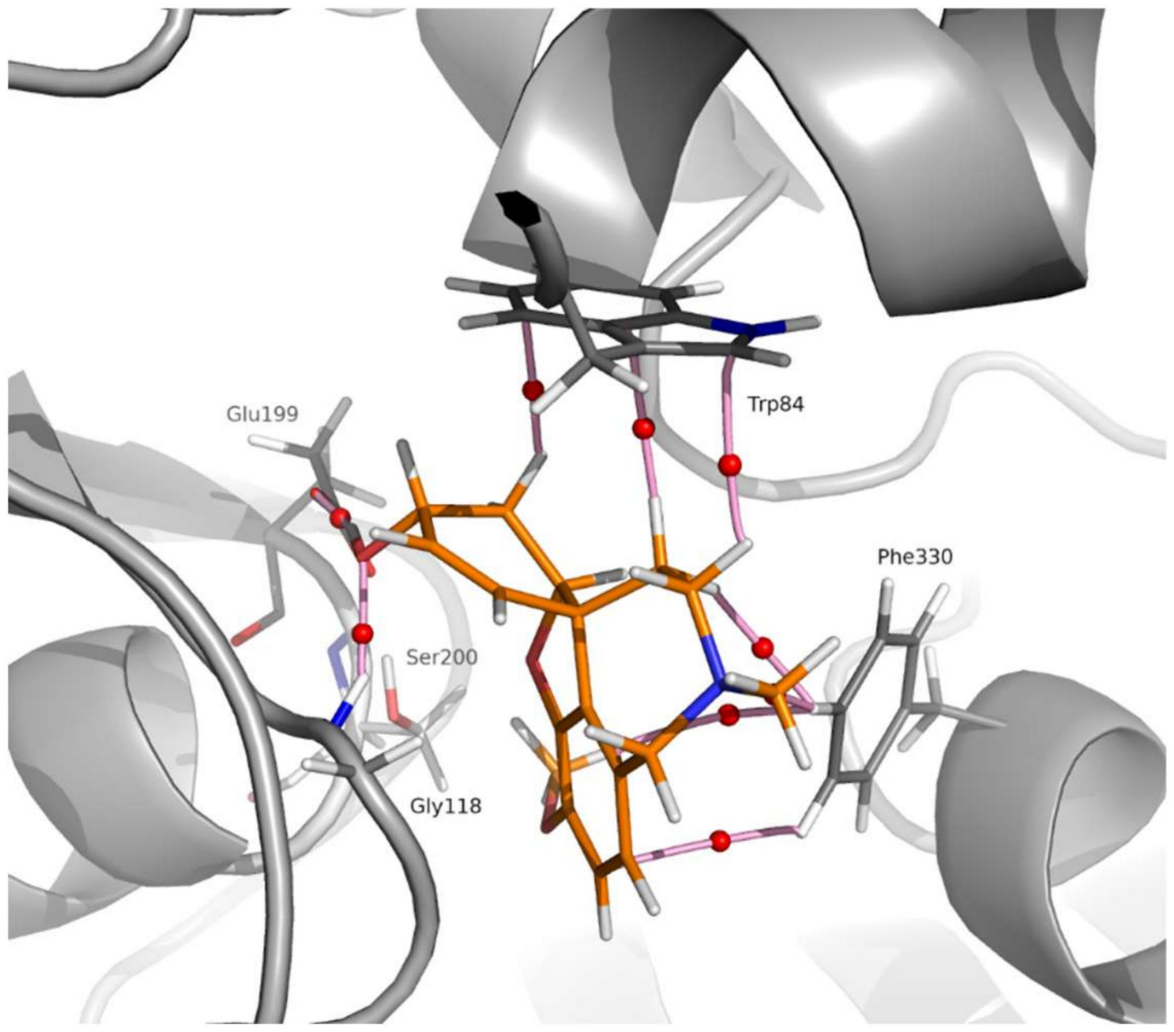
Molecular graph obtained for the non-covalent interactions between the main residues of AChE with galantamine (orange color). The structure of this complex was obtained from molecular dynamics simulations. The elements of the electron density topology are shown. The bond paths connecting the nuclei are represented in pink sticks and the bond critical points are shown as red spheres.

**Figure 4:**
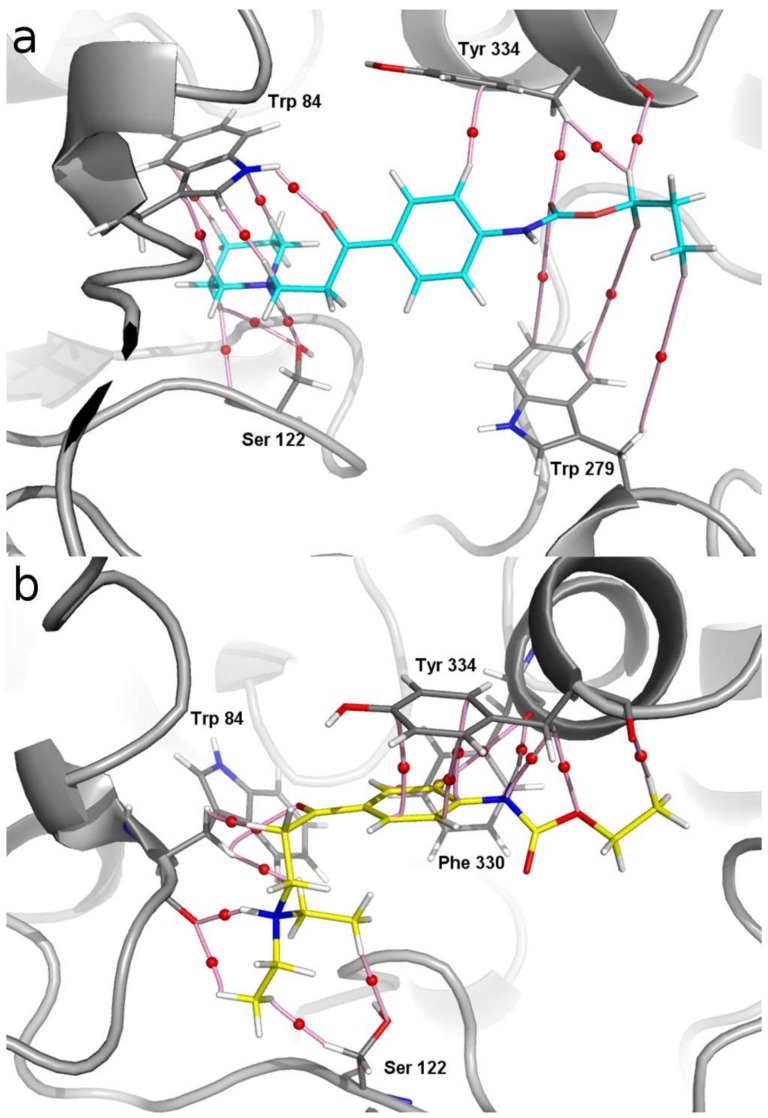
Molecular graph of the non-covalent interactions between the main residues of AChE with (**a**) compound **3** (light blue sticks) and (**b**) compound **6** (yellow sticks). The elements of the electron density topology are shown. The bond paths connecting the nuclei are represented in pink sticks and the bond critical points are shown as red spheres.

It is clear that more accurate calculations, such as quantum theory atoms in molecules (QTAIM) [30] analysis, are necessary for a detailed description of the interactions involved in the stabilization of the different complexes. The results are summarized in Figure 3 and Figure 4. It can be seen from Figure 3 that the main amino acids that stabilize the galantamine complex with AChE are: Trp84 ρ_(r)_ = 0.024 a.u. (atomic units, electronic density), Gly118 (0.052), Glu199 (0.061), Ser200 (0.013) and Phe330 (0.009). The respective ρ_(r)_ values obtained for each interaction indicate that such interactions are strong enough to stabilize very well this molecular complex. This result is in agreement with previously reported experimental and theoretical results [31,32].

Comparing the molecular graph of galantamine-AChE complex with that obtained for compound **3** (Figure 4a), it is possible to observe that the interactions with Trp84 and Ser122 are maintained. In fact, these are the strongest interactions, possessing values of ρ_(r)_ = 0.07 and 0.044 a.u., respectively. It should be noted that the interactions between the basic nitrogen of compound **3** with the OH group of Ser122 are strong hydrogen bonds. Other slightly weaker interactions that also help to stabilize this complex are those with Tyr334 and Trp279.

The molecular graph obtained for compound **2** is very similar to that of compound **3** and is shown in Figure S2 as Supplementary Material. In contrast to these results, the molecular graph obtained for compound **6** (Figure 4b) shows that only the interaction with Trp84 has been maintained in this case. In this complex the interaction with Ser122 is extremely weak with a very low value of ρ_(r)_ = 0.0175 a.u. Note that these O···HC interactions are a very weak type of non-classical hydrogen bond. From our results it appears that these complexes are stabilized mainly by π-π interactions stacking with Tyr334 as well as with interactions observed with Phe330. This is mainly due to the different spatial orientation adopted by the basic nitrogen atom when it is not part of the piperidine ring (compare Figure 4a,b). This is an important difference observed between the piperidine derivatives (compounds **1–3**) and the rest of the compounds reported here (compounds **4–16**). The different spatial ordering, as well as the different interactions observed for the piperidine derivatives with respect to the rest of the compounds reported here, could explain, at least in part, greater affinity that the compounds **1–3** have in comparison to the rest of the derivatives.

## 3. Conclusions

A series of novel arylaminopropanone hydrochloride salts with *N*-phenylcarbamate moiety **(1–16)** was synthesized and evaluated as potential AChE and BuChE inhibitors. In vitro experiments were performed with galantamine and rivastigmine as standards, which are the drugs currently used in the treatment of neurodegenerative diseases. Molecular modelling simulations were performed and compared with galantamine.

Three most potent agents, **1–3**, displayed inhibitory activity against AChE in vitro. Their percentage inhibition of AChE was comparable to galantamine and higher to rivastigmine, with IC_50_ in the micromolar range. Compound **3** was revealed as the most potent inhibitor, with an IC_50_ value of 6.57 µM comparable to the reference drug galantamine in the case of AChE. Also, compounds **1** and **2** expressed high activity with strong preferences for the AChE. The compounds with aliphatic substituents on basic *N* atom (compounds **6–16**) showed weak inhibitory activity. Only compounds **11**, **12** and **16** had percentage inhibition better than 50 %, but their IC_50_ values were higher than 100 µM.

The molecular modelling study indicates that compounds **1–16** bind to the same active site of AChE as galantamine. However, some caution must be taken with this statement since there is not the experimental evidence to corroborate it. Some of the molecular interactions obtained for the compounds reported here are a little weaker than those found for galantamine, which would indicate a possible lower affinity for the active site “binding pocket”. However, we observed some other strong molecular interactions which would indicate the binding to peripheral “anionic” active site. The differences in the activity of piperidine-substituted compounds **1–3** from alkyl-substituted compounds **6–16** can be explained by distinct spatial orientation of the basic *N* atom in amine closed in a ring-substructure and amine with alkyl chains.

The structure-activity relationship shows that the incorporation of a piperidine moiety (compounds **1–3**) leads to higher inhibitory activity. In comparison, the replacement of piperidine by morpholine significantly decreased the activity against AChE. The obtained results also revealed that compounds with longer alkylamine moiety, mainly compounds **10–13**, have a higher activity and selectivity to BuChE. The prolongation of alkyl chain in *N*-phenylcarbamate moiety leads to an increase in the activity.

Although the inhibitory activities of compounds **1–3** are slightly weaker than those of galantamine, these compounds show promising potential. They can be taken as starting structures for further molecular modelling and synthetic research.

## 4. Experimental Section

### 4.1. General Information

All chemicals and solvents are commercially available; they were purchased from Sigma-Aldrich or Lach-Ner, and were used without purification or after distillation and treatment with drying agents. The reactions and purity of compounds were monitored by thin-layer chromatography (TLC) on silica gel plates (60-F254 Merck) and visualized under UV light (254 nm). Melting points are uncorrected and they were determined on Kofler hot-plate apparatus HMK (Franz Kustner Nacht KG, Dresden, Germany).

The ^1^H and ^13^C NMR spectra were measured in DMSO-*d*_6_, chloroform-*d* or methanol-*d_4_* on a JNM-ECZ4OOR FT-NMR spectrometer 9.39 T (399.78 MHz for ^1^H and 100.53 MHz for ^13^C nucleus; Jeol Resonance, Tokyo, Japan) equipped with a 5 mm High Sensitivity PulseField Gradient Autotune™ probe. Chemical shifts are reported in ppm, referenced to the chemical shifts of residual solvent resonance (DMSO, 2.5 ppm for ^1^H and 39.5 ppm for ^13^C, chloroform 7.26 ppm for ^1^H and 77.0 ppm for ^13^C, methanol 3.31 for ^1^H and 49.15 ppm for ^13^C). The coupling constants (*J*) are reported in Hz. The spectra were recorded at a temperature of 30 °C.

High-resolution mass spectra were measured using a Dionex UltiMate^®^ 3000 high-performance liquid chromatograph (Termo-Fisher Scientifc, Waltham, MA, USA) coupled with a LTQ Orbitrap XLTM Hybrid Ion Trap-Orbitrap Fourier Transform Mass Spectrometer (Termo-Fisher Scientifc) with an injection into HESI II in the positive or negative mode.

The infrared (IR) spectra were measured on FT-IR spectrophotometer Nicolet Model Impact 410 using Smart ATR iD7 with the diamond crystal. The spectra were obtained by the accumulation of 64 scans in the region of 4,000–650 cm^−1^.

The in vitro testing of ChEs inhibition was performed on 96-well microplate reader, Synergy^TM^ HT, BioTek, Vinnoski, USA.

### 4.2. Synthesis

#### 4.2.1. Preparation of Carbamate Intermediates **1a–4a**

The appropriate alkyl chloroformate (40 mmol) in dichloromethane (5 mL) was dropwise added to a solution of 4-aminoacetophenone (40 mmol) in dichloromethane (20 mL) and pyridine (40 mmol). The mixture was stirred at room temperature for 2–6 h and the reaction progress was monitored by TLC, mobile phase toluene: acetone (2:1). After cooling in an ice bath, pyridinium chloride was filtered off and the solvent was evaporated under reduced pressure. The semi-solid product was recrystallized using propan-2-ol. For characterization data see [26].

#### 4.2.2. Preparation of Target Arylaminopropanones **1–16**

The mixture of carbamate (10 mmol), paraformaldehyde (50 mmol) and appropriate secondary amine (as a hydrochloride, 10 mmol) was suspended in tetrahydrofurane (40 mL). Then, hydrochloric acid (35%, 2 mmol) was added to the mixture. The mixture was stirred at reflux (70 °C) for 6–8 h and the reaction progress was monitored by TLC, mobile phase toluene: acetone (2:1). After cooling, the resulting white solids were collected by filtration. The final products, alkyl {4-[3-(alkylamino)propanoyl]phenyl}carbamate, were isolated from reaction mixture as hydrochlorides and recrystallized from propan-2-ol. The structures of target compounds are given in Scheme 1.

*1-(3-{4-[(methoxycarbonyl)amino]phenyl}-3-oxopropyl)piperidin-1-ium chloride* (**1**). White solid; Yield 73%; Mp 228–230 °C (HCl salt); ^1^H NMR (400 MHz, DMSO-d_6_) δ 10.54 (br. s, 1H, NH^+^), 10.13 (s, 1H, N**H**), 7.91–8.01 (m, 2H, Ar-C=O), 7.59–7.68 (m, 2H, Ar-NH), 3.70 (s, 3H, OC**H_3_**), 3.59 (t, *J* = 7.30 Hz, 2H, C(O)C**H_2_**), 3.41–3.52 (m, 2H), 3.32–3.38 (m, 2H, –C**H_2_**N^+^H), 2.83–2.98 (m, 2H), 1.74–1.83 (m, 4H), 1.65–1.74 (m, 1H), 1.30–1.47 (m, 1H); ^13^C NMR (101 MHz, DMSO-d_6_) δ 195.1, 153.7, 144.1, 130.0, 129.4, 117.3, 52.1, 51.9, 51.1, 32.3, 22.4, 21.3; HR-MS: for C_16_H_23_N_2_O_3_ [M+H]^+^ calculated 291.17032 *m/z*, found 291.17017 *m/z*; FT-IR (ATR); cm^−1^: 3021, 2944, 2534, 1717 (carbamate C=O), 1678 (ketone C=O), 1589, 1534, 1414, 1316, 1225, 1185, 1070, 962, 868, 768

*1-(3-{4-[(ethoxycarbonyl)amino]phenyl}-3-oxopropyl)piperidin-1-ium chloride* (**2**). White solid; Yield 62%; Mp 226–227 °C (HCl salt); ^1^H NMR (400 MHz, DMSO-d_6_) δ 10.56 (br. s., 1H, NH^+^), 10.12 (s, 1H, N**H**), 7.89–8.00 (m, 2H, Ar-C=O), 7.57–7.69 (m, 2H, Ar-NH), 4.16 (q, *J* = 7.32 Hz, 2H, OC**H_2_**), 3.54–3.65 (m, 2H, C(O)C**H_2_**), 3.43–3.50 (m, 2H), 3.27–3.36 (m, 2H, –C**H_2_**N^+^H), 2.82–3.01 (m, 2H), 1.63–1.82 (m, 5H), 1.31–1.45 (m, 1H), 1.26 (t, *J* = 7.09 Hz, 3H, OCH_2_C**H_3_**); ^13^C NMR (101 MHz, DMSO-d_6_) δ 195.1, 153.3, 144.3, 129.9, 129.4, 117.3, 60.6, 52.1, 51.2, 32.4, 22.4, 21.3, 14.4; HR-MS: for C_17_H_23_N_2_O_3_ [M-H]^-^ calculated 303.17142 *m/z*, found 303.17142 *m/z*; FT-IR (ATR); cm^−1^: 3030, 2944, 2531, 1717, 1677, 1588, 1534, 1416, 1315, 1224, 1186, 1062, 965, 867

*1-(3-oxo-3-{4-[(propoxycarbonyl)amino]phenyl}propyl)piperidin-1-ium chloride* (**3**). White solid; Yield 71%; Mp 209–211 °C (HCl salt); ^1^H NMR (400 MHz, DMSO-d_6_) δ 10.49 (br. s., 1H, NH^+^), 10.13 (s, 1H, N**H**), 7.92–7.99 (m, 2H, Ar-C=O), 7.60–7.66 (m, 2H, Ar-NH), 4.07 (t, *J* = 6.63 Hz, 2H, OC**H_2_**), 3.59 (t, *J* = 7.30 Hz, 2H, C(O)C**H_2_**), 3.42–3.50 (m, 2H), 3.27–3.34 (m, 2H, –C**H_2_**N^+^H), 2.82–2.99 (m, 2H), 1.57–1.87 (m, 7H), 1.29–1.45 (m, 1H), 0.94 (t, *J* = 7.30 Hz, 3H, OCH_2_CH_2_C**H_3_**); ^13^C NMR (101 MHz, DMSO-d_6_) δ 195.1, 153.4, 144.3, 129.9, 129.4, 117.3, 66.1, 52.1, 51.2, 32.4, 22.4, 21.8, 21.3, 10.2; HR-MS: for C_17_H_25_N_2_O_3_ [M-H]^-^ calculated 317.18707 *m/z*, found 317.18741 *m/z*; FT-IR (ATR); cm^−1^: 3027, 2931, 2530, 1711, 1676, 1587, 1413, 1314, 1219, 1183, 1058, 964, 857, 797

*4-(3-{4-[(ethoxycarbonyl)amino]phenyl}-3-oxopropyl)morpholin-4-ium chloride* (**4**). White solid; Yield 71%; Mp 209–211 °C (HCl salt); ^1^H NMR (400 MHz, DMSO-d_6_) δ 11.32 (br. s., 1H, NH^+^), 10.09 (s, 1H, N**H**), 7.90–7.99 (m, 2H, Ar-C=O), 7.59–7.68 (m, 2H, Ar-NH), 4.16 (q, *J* = 6.86 Hz, 2H, OC**H_2_**), 3.89–4.02 (m, 2H, CH_2_OCH_2_) 3.75–3.88 (m, 2H, CH_2_OCH_2_), 3.56–3.66 (m, 2H, C(O)C**H_2_**), 3.37–3.53 (m, 4H, –C**H_2_**N^+^H), 3.01–3.21 (m, 2H), 1.26 (t, *J* = 7.09 Hz, 3H, OCH_2_C**H_3_**); ^13^C NMR (101 MHz, DMSO-d_6_) δ 194.9, 153.3, 144.2, 129.9, 129.4, 117.3, 63.2, 60.5, 51.2, 51.1, 32.0, 14.3; HR-MS: for C_16_H_21_N_2_O_4_ [M-H]^-^ calculated 305.15068 *m/z*, found 305.15091 *m/z*; FT-IR (ATR); cm^−1^: 3538, 3160, 3030, 2530, 2456, 1714, 1675, 1587, 1531, 1412, 1313, 1183, 1065, 973, 859, 759

*4-(3-oxo-3-{4-[(propoxycarbonyl)amino]phenyl}propyl)morpholin-4-ium chloride* (**5**). White solid; Yield 71%; Mp 164–169 °C (HCl salt); ^1^H NMR (400 MHz, DMSO-d_6_) δ 11.22 (br. s., 1H, NH^+^), 10.09 (s, 1H, N**H**), 7.89–8.02 (m, 2H, Ar-C=O), 7.57–7.70 (m, 2H, Ar-NH), 4.07 (t, *J* = 6.57 Hz, 2H, OC**H_2_**), 3.91–4.03 (m, 2H, CH_2_OCH_2_), 3.73–3.87 (m, 2H, CH_2_OCH_2_), 3.54–3.66 (m, 2H, C(O)C**H_2_**), 3.37–3.53 (m, 4H, –C**H_2_**N^+^H), 3.02–3.20 (m, 2H), 1.66 (sxt, *J* = 7.12 Hz, 2H, OCH_2_C**H_2_**CH_3_), 0.94 (t, *J* = 7.43 Hz, 3H, OCH_2_CH_2_C**H_3_**); ^13^C NMR (101 MHz, DMSO-d_6_) δ 195.0, 153.4, 144.3, 129.9, 129.5, 117.3, 66.1, 63.2, 51.2, 51.2, 32.1, 21.8, 10.3; HR-MS: for C_17_H_23_N_2_O_4_ [M-H]^-^ calculated 319.16633 *m/z*, found 319.16644 *m/z*; FT-IR (ATR); cm^−1^: 3316, 2969, 2585, 2361, 1734, 1665, 1587, 1528, 1418, 1317, 1212, 1187, 1119, 969, 854, 766

*(3-{4-[(ethoxycarbonyl)amino]phenyl}-3-oxopropyl)diethylazanium chloride* (**6**). White solid; Yield 36%; Mp 141–143 °C (HCl salt); ^1^H NMR (DMSO-d_6_, 400 MHz): δ 10.58 (br. s., 1 H, NH^+^), 10.13 (s, 1 H, N**H**), 7.94–7.99 (m, 2 H, Ar-C=O), 7.60 - 7.67 (m, 2 H, Ar-NH), 4.16 (q, *J*=7.3 Hz, 2 H, OC**H_2_**), 3.55 (t, *J*=7.3 Hz, 2 H, C(O)C**H_2_**), 3.30–3.40 (m, 2 H, –C**H_2_**N^+^), 3.10–3.20 (m, 4 H), 1.18–1.29 ppm (m, 9 H, OCH_2_C**H_3_**; N(CH_2_C**H_3_**)_2_); ^13^C NMR (101 MHz, DMSO-d_6_) δ 195.1, 153.3, 144.3, 130.0, 129.5, 117.2, 60.6, 46.2, 45.9, 32.0, 14.4, 8.5; HR-MS: for C_16_H_25_N_2_O_3_ [M+H]^+^ calculated 293.18597 *m/z*, found 293.18573 *m/z*; FT-IR (ATR); cm^−1^: 3321, 2987, 2590, 2483, 1713, 1663, 1589, 1535, 1420, 1319, 1220, 1189, 1064, 963, 769

*Butyl(3-{4-[(methoxycarbonyl)amino]phenyl}-3-oxopropyl)methylazanium chloride* (**7**). White solid; Yield 75%; Mp 147–149 °C (HCl salt); ^1^H NMR (400 MHz, DMSO-d_6_) Shift 10.37 (br. s., 1H, NH^+^), 10.10 (s, 1H, N**H**), 7.93–8.00 (m, 2H, Ar-C=O), 7.60–7.67 (m, 2H, Ar-NH), 3.70 (s, 3H, OC**H_3_**), 3.50–3.60 (m, 2H, C(O)C**H_2_**), 3.41–3.49 (m, 1H, –C**H_2_**N^+^), 3.30–3.38 (m, 1H, –C**H_2_**N^+^), 2.96–3.21 (m, 2H), 2.76 (d, J = 4.57 Hz, 3H, N^+^C**H_3_**), 1.62–1.73 (m, 2H, N^+^CH_2_C**H_2_**CH_2_CH_3_), 1.27–1.41 (m, 2H, N^+^CH_2_CH_2_C**H_2_**CH_3_), 0.92 (t, J = 7.32 Hz, 3H, 3H, N^+^CH_2_CH_2_CH_2_C**H_3_**); ^13^C NMR (101 MHz, DMSO-d_6_) δ 195.2, 153.8, 144.2, 130.0, 129.5, 117.3, 54.8, 52.0, 50.2, 38.9, 32.3, 25.3, 19.4, 13.5; HR-MS: for C_16_H_23_N_2_O_3_ [M-H]^-^ calculated 291.17142 *m/z*, found 291.17172 *m/z*; FT-IR (ATR); cm^−1^: 3304, 2958, 2593, 2503, 1732, 1665, 1590, 1537, 1419, 1321, 1220, 1189, 1069, 985, 851

*Butyl(3-{4-[(ethoxycarbonyl)amino]phenyl}-3-oxopropyl)methylazanium chloride* (**8**). White solid; Yield 53%; Mp 146–148 °C (HCl salt); ^1^H NMR (400 MHz, DMSO-d_6_) δ 10.46 (br. s., 1H, NH^+^), 10.09 (s, 1H, N**H**), 7.92–8.00 (m, 2H, Ar-C=O), 7.59–7.67 (m, 2H, Ar-NH), 4.16 (q, *J* = 6.86 Hz, 2H, OC**H_2_**), 3.52–3.63 (m, 2H, C(O)C**H_2_**), 3.39–3.49 (m, 1H, –C**H_2_**N^+^), 3.32–3.37 (m, 1H, –C**H_2_**N^+^), 3.09–3.20 (m, 1H, N^+^C**H_2_**), 2.96–3.08 (m, 1H, N^+^C**H_2_**), 2.76 (d, *J* = 4.57 Hz, 3H, NC**H_3_**), 1.60–1.73 (m, 2H, N^+^CH_2_C**H_2_**CH_2_CH_3_), 1.29–1.39 (m, 2H, N^+^CH_2_CH_2_C**H_2_**CH_3_), 1.26 (t, *J* = 7.09 Hz, 3H, OCH_2_C**H_3_**), 0.92 (t, *J* = 7.32 Hz, 3H, NCH_2_CH_2_CH_2_C**H_3_**); ^13^C NMR (101 MHz, DMSO-d_6_) δ 195.1, 153.3, 144.2, 129.9, 129.4, 117.2, 60.5, 54.8, 50.2, 38.9 32.3, 25.3, 19.3, 14.4, 13.4; HR-MS: for C_17_H_25_N_2_O_3_ [M-H]^-^ calculated 305.18707 *m/z*, found 305.18738 *m/z*; FT-IR (ATR); cm^−1^: 3318, 2961, 2925, 2586, 2497, 1720, 1663, 1588, 1534, 1419, 1319, 1217, 1092, 1063, 899, 850, 767

*Butyl(methyl)(3-oxo-3-{4-[(propoxycarbonyl)amino]phenyl}propyl)azanium chloride* (**9**). White solid; Yield 43%; Mp 142–144 °C (HCl salt); ^1^H NMR (400 MHz, DMSO-d_6_) δ 10.51 (br. s., 1H, NH^+^), 10.11 (s, 1H, N**H**), 7.92–8.01 (m, 2H, Ar-C=O), 7.60–7.68 (m, 2H, Ar-NH), 4.07 (t, *J* = 6.63 Hz, 2H, OC**H_2_**), 3.52–3.61 (m, 2H, C(O)C**H_2_**), 3.39–3.50 (m, 1H, –C**H_2_**N^+^), 3.26–3.33 (m, 1H, –C**H_2_**N^+^), 2.96–3.20 (m, 2H, N^+^C**H_2_**), 2.76 (d, *J* = 5.03 Hz, 3H, NC**H_3_**), 1.59–1.74 (m, 4H, N^+^CH_2_C**H_2_**CH_2_CH_3_ and OCH_2_C**H_2_**), 1.26–1.40 (m, 2H, N^+^CH_2_CH_2_C**H_2_**CH_3_), 0.87–1.00 (m, 6H, OCH_2_CH_2_C**H_3_;** NCH_2_CH_2_CH_2_C**H_3_**); ^13^C NMR (101 MHz, DMSO-d_6_) δ 195.1, 153.4, 144.2, 129.9, 129.4, 117.2, 66.0, 54.8, 50.2, 38.9, 32.3, 25.3, 21.8, 19.4, 13.5, 10.2; HR-MS: for C_18_H_27_N_2_O_3_ [M-H]^-^ calculated 319.20272 *m/z*, found 319.20279 *m/z*; FT-IR (ATR); cm^−1^: 3311, 2965, 2939, 2877, 2454, 1721, 1669,1591, 1534, 1420, 1320, 1220, 1189, 1092, 1061, 897, 852, 787

*Butyl(ethyl)(3-{4-[(methoxycarbonyl)amino]phenyl}-3-oxopropyl)azanium chloride* (**10**). White solid; Yield 35%; Mp 119–122 °C (HCl salt); ^1^H NMR (400 MHz, DMSO-d_6_) δ 10.54 (br. s., 1H, NH^+^), 10.17 (s, 1H, N**H**), 7.95–8.00 (m, 2H, Ar-C=O), 7.61–7.66 (m, 2H), 3.69 (s, 3H, OC**H_3_**), 3.51–3.60 (m, 2H, C(O)C**H_2_**), 3.35–3.44 (m, 2H), 3.12–3.21 (m, 2H), 3.00–3.11 (m, 2H), 1.59–1.72 (m, 2H, N^+^CH_2_C**H_2_**CH_2_CH_3_), 1.28 - 1.39 (m, 2H, N^+^CH_2_CH_2_C**H_2_**CH_3_), 1.24 (t, *J* = 7.09 Hz, 3H, NCH_2_C**H_3_**), 0.91 (t, *J* = 7.30 Hz, 3H, NCH_2_CH_2_CH_2_C**H_3_**); ^13^C NMR (101 MHz, DMSO-d_6_) δ 195.1, 153.8, 144.2, 130.1, 129.5, 117.2, 52.0, 51.2, 46.9, 46.5, 32.0, 25.0, 19.5, 13.6, 8.5; HR-MS: for C_17_H_25_N_2_O_3_ [M-H]^-^ calculated 305.18707 *m/z*, found 305.18726 *m/z*; FT-IR (ATR); cm^−1^: 3307, 2960, 2875, 2491, 1727, 1663, 1589, 1538, 1419, 1321, 1221, 1189, 1071, 899

*Butyl(3-{4-[(ethoxycarbonyl)amino]phenyl}-3-oxopropyl)ethylazanium chloride* (**11**). White solid; Yield 68%; Mp 142–145 °C (HCl salt); 1H NMR (400 MHz, CHLOROFORM-d) Shift 12.08 (br. s., 1H, N^+^H), 7.85–7.96 (m, 2H, Ar-C=O), 7.53–7.61 (m, 2H, Ar-NH), 7.51 (s, 1H, NH), 4.22 (q, J = 7.32 Hz, 2H, OC**H_2_**), 3.70 (t, J = 7.32 Hz, 2H, C(O)C**H_2_**), 3.39–3.50 (m, 2H, –C**H_2_**N^+^), 2.94–3.24 (m, 4H, N^+^(C**H_2_**)_2_), 1.67–1.90 (m, 2H, N^+^CH_2_C**H_2_**CH_2_CH_3_), 1.35–1.46 (m, 5H, N^+^CH_2_CH_2_C**H_2_**CH_3_ and N^+^CH_2_C**H_3_**), 1.30 (t, J = 7.09 Hz, 3H, OCH_2_C**H_3_**), 0.95 (t, J = 7.32 Hz, 3H, NCH_2_CH_2_CH_2_C**H_3_**); ^13^C NMR (101 MHz, CHLOROFORM-d) δ 194.5, 153.1, 143.6, 130.1, 129.8, 117.7, 61.6, 51.7, 47.8, 47.3, 33.1, 24.8, 20.1, 14.5, 13.5, 8.3; HR-MS: for C_18_H_27_N_2_O_3_ [M-H]^-^ calculated 319.20272 *m/z*, found 319.20285 *m/z*; FT-IR (ATR); cm^−1^: 3321, 2959, 2935, 2438, 1726, 1662, 1589, 1533, 1420, 1318, 1219, 1090, 1062, 981, 770

*Butyl(ethyl)(3-{4-[(propoxycarbonyl)amino]phenyl}-3-oxopropyl)azanium chloride* (**12**). White solid; Yield 45%; Mp 154–156 °C (HCl salt); ^1^H NMR (400 MHz, DMSO-d_6_) δ 10.58 (br. s., 1H, NH^+^), 10.11 (s, 1H, N**H**), 7.93–8.00 (m, 2H, Ar-C=O), 7.60–7.66 (m, 2H, Ar-NH), 4.07 (t, *J* = 6.63 Hz, 2H, OC**H_2_**), 3.51–3.59 (m, 2H, C(O)C**H_2_**), 3.35–3.41 (m, 2H, –C**H_2_**N^+^), 3.12–3.21 (m, 2H), 3.01–3.10 (m, 2H), 1.60–1.71 (m, 4H, N^+^CH_2_C**H_2_**CH_2_CH_3_ and OCH_2_C**H_2_**), 1.28–1.39 (m, 2H, N^+^CH_2_CH_2_C**H_2_**CH_3_), 1.24 (t, *J* = 7.32 Hz, 3H, NCH_2_C**H_3_**), 0.88–0.97 (m, 6H, OCH_2_CH_2_C**H_3_**; NCH_2_CH_2_CH_2_C**H_3_**); ^13^C NMR (101 MHz, DMSO-d_6_) δ 195.1, 153.4, 144.2, 130.0, 129.4, 117.2, 66.0, 51.1, 46.9, 46.5, 31.9, 24.9, 21.8, 19.4, 13.5, 10.2, 8.4; HR-MS: for C_19_H_29_N_2_O_3_ [M-H]^-^ calculated 333.21837 *m/z*, found 333.21869 *m/z*; FT-IR (ATR); cm^−1^: 3323, 2967, 2937, 2479, 1721, 1661, 1593, 1534, 1421, 1421, 1322, 1222, 1191, 1061, 978

*(3-{4-[(butoxycarbonyl)amino]phenyl}-3-oxopropyl)(butyl)ethylazanium chloride* (**13**). White solid; Yield 50%; Mp 135–138 °C (HCl salt); ^1^H NMR (400 MHz, DMSO-d_6_) δ 10.44 (br. s., 1H, NH^+^), 10.10 (s, 1H, N**H**), 7.93–7.99 (m, 2H, Ar-C=O), 7.60–7.66 (m, 2H, Ar-NH), 4.11 (t, *J* = 6.63 Hz, 2H, OC**H_2_**), 3.50–3.59 (m, 2H, C(O)C**H_2_**), 3.34–3.42 (m, 2H, –C**H_2_**N^+^), 3.11–3.22 (m, 2H), 3.00–3.11 (m, 2H), 1.56–1.72 (m, 4H, N^+^CH_2_C**H_2_**CH_2_CH_3_ and OCH_2_C**H_2_**), 1.28–1.44 (m, 4H, N^+^CH_2_CH_2_C**H_2_**CH_3_ and OCH_2_CH_2_C**H_2_**CH_3_), 1.24 (t, *J* = 7.32 Hz, 3H, N^+^CH_2_C**H_3_**), 0.88–0.95 (m, 6H, OCH_2_CH_2_CH_2_C**H_3_**; NCH_2_CH_2_CH_2_C**H_3_**); ^13^C NMR (101 MHz, DMSO-d_6_) δ 195.1, 153.4, 144.2, 130.0, 129.4, 117.2, 64.3, 51.2, 46.9, 46.5, 31.9, 30.4, 24.9, 19.4, 18.5, 13.5, 13.5, 8.4; HR-MS: for C_20_H_31_N_2_O_3_ [M-H]^-^ calculated 347.23402 *m/z*, found 347.23416 *m/z*; FT-IR (ATR); cm^−1^: 3319, 2960, 2926, 2874, 2459, 1721, 1661, 1592, 1536, 1420, 1319, 1218, 1090, 897

*(3-{4-[(methoxycarbonyl)amino]phenyl}-3-oxopropyl)(methyl)propylazanium chloride* (**14**). White solid; Yield 75%; Mp 172–175 °C (HCl salt); ^1^H NMR (400 MHz, METHANOL-d_4_) δ 7.96–8.05 (m, 2H, Ar-C=O), 7.57–7.67 (m, 2H, Ar-NH), 3.77 (s, 3H, OC**H_3_**), 3.65–3.73 (m, 1H, –C**H_2_**N^+^), 3.54–3.61 (m, 2H, C(O)C**H_2_**), 3.37–3.46 (m, 1H, –C**H_2_**N^+^), 3.08–3.26 (m, 2H), 2.91 (s, 3H, N^+^C**H_3_**), 1.75–1.94 (m, 2H, N^+^CH_2_C**H_2_**CH_3_), 1.06 (t, *J* = 7.55 Hz, 3H, N^+^CH_2_CH_2_C**H_3_**); ^13^C NMR (101 MHz, METHANOL-d_4_) δ 197.0, 156.1, 146.1, 131.6, 130.9, 118.8, 59.8, 52.9, 52.8, 41.2, 33.7, 18.9, 11.3; HR-MS: for C_15_H_21_N_2_O_3_ [M-H]^-^ calculated 277.15577 *m/z*, found 277.15601 *m/z*; FT-IR (ATR); cm^−1^: 3306, 2966, 2948, 2590, 2468, 1725, 1665, 1589, 1537, 1418, 1321, 1220, 1189, 1070, 975, 850

*(3-{4-[(ethoxycarbonyl)amino]phenyl}-3-oxopropyl)(methyl)propylazanium chloride* (**15**). White solid; Yield 58%; Mp 144–147 °C (HCl salt); ^1^H NMR (400 MHz, DMSO-d_6_) δ 10.07 (s, 1H, N**H**), 7.88 - 7.98 (m, 2H, Ar-C=O), 7.57–7.65 (m, 2H, Ar-NH), 4.16 (q, *J* = 7.32 Hz, 2H), 3.27–3.48 (m, 4H, C(O)C**H_2_** and C**H_2_**N^+^), 3.01–3.17 (m, 2H), 2.66–2.83 (m, 3H), 1.50–1.68 (m, 2H, N^+^CH_2_C**H_2_**), 1.26 (t, *J* = 7.09 Hz, 3H, OCH_2_C**H_3_**), 0.88 (t, *J* = 7.30 Hz, 3H, N^+^CH_2_CH_2_C**H_3_**); ^13^C NMR (101 MHz, DMSO-d_6_) δ 196.3, 153.3, 144.0, 130.3, 129.3, 117.2, 60.5, 57.4, 51.1, 40.3, 33.6, 18.1, 14.4, 11.2; HR-MS: for C_16_H_23_N_2_O_3_ [M-H]^-^ calculated 291.17142 *m/z*, found 291.17178 *m/z*; FT-IR (ATR); cm^−1^: 3316, 2971, 2420, 1711, 1664, 1590, 1532, 1419, 1318, 1219, 1189, 1061, 976, 848, 770

*(3-{4-[(ethoxycarbonyl)amino]phenyl}-3-oxopropyl)(methyl)pentylazanium chloride* (**16**). White solid; Yield 48%; Mp 120–123 °C (HCl salt); ^1^H NMR (400 MHz, METHANOL-d_4_) δ 7.97–8.04 (m, 2H, Ar-C=O), 7.57–7.65 (m, 2H, Ar-NH), 4.21 (q, *J* = 7.32 Hz, 2H, OC**H_2_**), 3.63–3.78 (m, 1H, –C**H_2_**N^+^), 3.52–3.62 (m, 2H, C(O)C**H_2_**), 3.35–3.47 (m, 1H, –C**H_2_**N^+^), 3.26 (t, *J* = 8.20 Hz, 1H, N^+^C**H_2_**), 3.17 (t, *J* = 8.20 Hz, 1H, N^+^C**H_2_**), 2.90 (s, 3H, NC**H_3_**), 1.76–1.88 (m, 2H, N^+^CH_2_C**H_2_**), 1.36–1.50 (m, 4H), 1.32 (t, *J* = 7.09 Hz, 3H, OCH_2_C**H_3_**), 0.97 (t, *J* = 6.90 Hz, 3H, N-alkyl-C**H_3_**); ^13^C NMR (101 MHz, METHANOL-d_4_) δ 197.1, 155.7, 146.2, 131.6, 130.9, 118.9, 62.4, 58.4, 52.8, 41.2, 33.7, 29.8, 25.2, 23.4, 15.0, 14.3; HR-MS: for C_18_H_27_N_2_O_3_ [M-H]^-^ calculated 319.20272 *m/z*, found 319.20322 *m/z*; FT-IR (ATR); cm^−1^: 3324, 2951, 2589, 2508, 1711, 1662, 1590, 1535, 1483, 1421, 1320, 1223, 1190, 1064, 985, 852, 785

### 4.3. Enzyme Assays

The method originally developed by Ellman was used with slight modifications [27]. The measurement of AChE and BuChE inhibitory activity was performed in a 96-well microplate reader. 170 μL of 0.1 M phosphate buffer (pH 7.0), 20 μL of AChE (electric eel) or BuChE (equine serum) in phosphate solution (2.3 U/mL), 20 μL of the tested compound in methanol and 20 μL of 10 mM (5,5′-dithiobis(2-nitrobenzoic acid), DTNB, Ellman’s Reagent) were mixed and incubated at 37 °C for 15 min.

Then 20 μL of 7.5 mM acetylthiocholine iodide (ATCI)/butyrylthiocholine iodide (BTCI) were added. The absorbance was measured at 405 nm (Abs_sample_). Each measurement was repeated five times (n = 5). There was prepared one set of mixtures as a control (Abs_control_), which contained an equal volume of methanol instead of the tested samples.

The percentage ability to inhibit AChE and BuChE at the concentration 100 µM was tested for all compounds. The inhibitory rates IR (%) were calculated according to the Equation (1).

IR = [1 - (Abs_sample_/Abs_control_)] × 100 (1)

As a second step, the compounds that exhibited inhibitory potency higher than 50 % at the concentration of 100 µM were chosen for the determination of IC_50_ values. These compounds were used in concentrations of 1.6, 3.2, 6.3, 12.5, 25, 50 μM for IC_50_ determination, each concentration was measured three times (n = 3). The values were calculated by non-linear regression of the concentration-response curve using GraphPad Prism 5.0 software, according to the Equation (2):

Y = Y_min_ + (Y_max_ − Y_min_)/(1 + [(X^HillSlope^)/(IC_50_^HillSlope^)]) (2)

### 4.4. Molecular Modelling

#### 4.4.1. Molecular Modelling

For our studies, we used the X-ray structure of the acetylcholinesterase *Torpedo californica* available on the Protein Data Bank (http://www.rcsb.org; code PDB: 1DX6) [31]. The water and ligand molecules were removed from the PDB structure before calculations.

#### 4.4.2. Molecular Docking

AutoDock version 4.0 [28] was used in our simulations. During the docking procedures, water molecules and ligands were removed from the protein. The receptor structure was defined as rigid. The grid dimensions were 60, 60, and 60 for the X, Y, and Z axes, respectively, in the active site of the AChE with the resolution of 0.375 Å. Gasteiger charges were assigned for all the compounds and nonpolar hydrogen atoms were merged. All torsions of the ligand were allowed to rotate during docking. AutoDock Tools 1.5.4 were used for all graphic manipulations and visualizations and ligand docking with the AutoDock version 4.0 [28]. The docking results were clustered on the basis of root-mean square deviation (rmsd) between the Cartesian coordinates of the ligand atoms and were ranked according to the binding free energy. As the optimum docking conformations were chosen those with the relative lower binding free energy and more populations.

#### 4.4.3. Molecular Dynamics (MD) Simulations

A series of MD simulations were carried out for all complexes obtained under the docking procedures. The simulations and the analysis of the trajectories were performed with Amber 12 package [29] using the FF99SB force field [33]. The complexes were constructed using the Leap module, minimized by the sander module, and the ‘‘pmemd’’ module was used for MD simulations. The ligand parameters were estimated with the antechamber module, based on the general AMBER force field (GAFF) [34]. Na^+^ ions were added to neutralize the charge of the system. The complexes were subsequently solvated in a truncated octahedral periodic box using the TIP3P water model [35], with a margin of 10.0 Å in each direction from the solute. The systems were then equilibrated by a 500 ps of energy minimization with position restraints on the protein and ligand to allow for relaxation of the solvent molecules. The equilibration run was followed by a 10 ns MD run without position restraints under periodic boundary conditions.

The non-bonded list was generated using an atom-based cutoff of 8.0 Å. The electrostatic term was described with the particle mesh Ewald algorithm [36]. The time step of the MD simulations was set to 2.0 fs, and the SHAKE algorithm [37] was used to constrain bond lengths at their equilibrium values. All the production simulations were performed under NPT conditions. The temperature was maintained at 298 K using the Langevin thermostat [38] with collision frequency of 1 ps^−1^. The coordinates were saved for analyses every 10 ps. The analysis of the trajectories was performed using AMBER analysis tools [19] and PyMOL [39]. This procedure was similar to that previously reported in the reference [40].

#### 4.4.4. Binding Energy Calculations

The interactions between the inhibitors and each residue of AChE were calculated using the MM/GBSA decomposition program implemented in AMBER 12. The interaction between inhibitor-residue pairs is approximated by the Equation (3):

ΔGA*_inh-residue_*− residue = ΔG*_vdw_* + ΔG*_ele_* + ΔG*_GB_* + ΔG*_SA_* (3)

where ΔG*_vdw_* and ΔG*_ele_* are non-bonded van der Waals interactions and electrostatic interactions between the inhibitors and each paratope residue in the gas phase. The polar contribution to solvation free energy (ΔG*_GB_*) was calculated by using the GB module [41]. ΔG*_SA_* is free energy due to the solvation process of non-polar contribution and was calculated from SASA. All energy components in Equation 3 were calculated using 500 snapshots from the last 5 ns of the MD simulation.

#### 4.4.5. Atoms in Molecules Theory

The quantum-mechanics calculations were used to complete the molecular modelling study. For this purpose, a reduced model of the active site of the enzyme was used. The wave functions of the inhibitors bound to the binding site residues (residues that have at least one heavy atom within 5 Å from the ligand molecule (first shell residues)), generated at the M062X/6-31G(d) level of theory, were subjected to the Quantum Theory Atoms In Molecules (QTAIM) analysis [30] using Multiwfn software [42]. The topological properties of a scalar field such as ρ_(r)_ are summarized in terms of their critical points, i.e., the points rc where Δρ_(r)_ = 0. Critical points are classified according to their type (ω, σ) by stating their rank, ω, and signature, σ. The rank is equal to the number of nonzero eigenvalues of the Hessian matrix of ρ(r) at rc, while the signature is the algebraic sum of the signs of the eigenvalues of this matrix. Critical points of (3, −1) and (3, +1) type describe saddle points, while the (3, −3) is a maximum and (3, +3) is a minimum in the field. Among these critical points, the (3, −1) or bond critical points are the most relevant ones since they are found between any two atoms linked by a chemical bond. This type of calculation was used in recent works because it ensures a reasonable compromise between the wave function quality required to obtain reliable values of the derivatives of ρ_(r)_ and the computer power available, due to the extension of the systems in the study [43,44,45,46,47].

## Figures and Tables

**Table 1:** Inhibitory activities of target compounds **1–16** against acetylcholinesterase (AChE) and butyrylcholinesterase (BuChE) in comparison with the standards galantamine and rivastigmine.

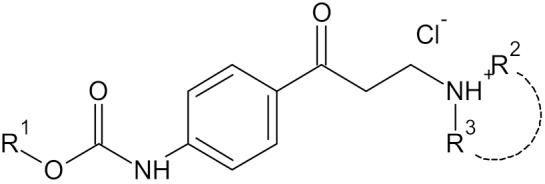							
compound	R^1^	R^2^	R^3^	AChE^a^ (%)	AChE IC_50_ (µM)	BuChE^a^ (%)	BuChE IC_50_ (µM)
**1**	Me	piperidine		74.1 ± 0.9	12.7	10.0 ± 0.8	>100
**2**	Et	piperidine		77.8 ± 0.4	8.58	15.7 ± 1.9	>100
**3**	Pr	piperidine		79.4 ± 0.3	6.57	21.0 ± 2.5	>100
**4**	Et	morpholine		16.1 ± 4.2	>100	17.8 ± 3.4	>100
**5**	Pr	morpholine		25.9 ± 3.2	>100	14.4 ± 2.4	>100
**6**	Et	Et	Et	42.2 ± 4.3	>100	34.2 ± 2.6	>100
**7**	Me	Bu	Me	38.0 ± 4.2	>100	20.4 ± 5.8	>100
**8**	Et	Bu	Me	23.5 ± 2.2	>100	4.7 ± 3.0	>100
**9**	Pr	Bu	Me	44.5 ± 1.1	>100	29.0 ± 2.9	>100
**10**	Me	Bu	Et	23.6 ± 2.3	>100	43.6 ± 3.0	>100
**11**	Et	Bu	Et	24.6 ± 1.9	>100	51.0 ± 2.9	>100
**12**	Pr	Bu	Et	38.4 ± 1.3	>100	51.1 ± 3.3	>100
**13**	Bu	Bu	Et	33.5 ± 3.4	>100	48.7 ± 2.6	>100
**14**	Me	Pr	Me	42.2 ± 1.3	>100	22.3 ± 4.6	>100
**15**	Et	Pr	Me	45.3 ± 3.4	>100	36.8 ± 2.1	>100
**16**	Et	Pe	Me	50.6 ± 1.8	>100	46.3 ± 2.0	>100
**Galantamine**				87.9 ± 0.5	1.1	62.1 ± 1.4	73.6
**Rivastigmine**				54.8 ± 4.4	87.9	72.9 ± 1.5	11.6

^a^ Percentage of inhibition at 100 μM expressed as the mean ± SD (n = 5)

## Supplementary Materials

The following are available online. Figure S1: Histogram of compound **2**; Figure S2: Molecular graph of compound **2.**
