# Supplementary material for: Arylaminopropanone Derivatives as Potential Cholinesterase Inhibitors: Synthesis, Docking Study and Biological Evaluation
Source: Molecules. 2020 Apr 10;25(7):1751. doi: 10.3390/molecules25071751 (PMC7180927; doi:10.3390/molecules25071751)
Supplement: Supplementary file 1 [file molecules-25-01751-s001.pdf]

SUPPLEMENTARY MATERIALS

Arylaminopropanone derivatives as potential cholinesterase inhibitors: Synthesis, docking study and biological evaluation

Anna Hudcová, Aleš Kroutil, Renata Kubínová, Adriana D. Garro, Lucas J. Gutierrez, Ricardo D. Enriz, Michal Oravec and Jozef Csöllei

Fig. S 1: Histogram of compound **2**
Fig. S 2: Molecular graph of compound **2**
Fig. S 3: The distances [Å] for the center of mass of molecules **3** and **6** with respect to galantamine.
Fig. S 4: ^{1}H NMR spectrum of compound **1**
Fig. S 5: ^{13}C NMR spectrum of compound **1**
Fig. S 6: ^{1}H NMR spectrum of compound **2**
Fig. S 7: ^{13}C NMR spectrum of compound **2**
Fig. S 8: ^{1}H NMR spectrum of compound **3**
Fig. S 9: ^{13}C NMR spectrum of compound **3**
Fig. S 10: ^{1}H NMR spectrum of compound **4**
Fig. S 11: ^{1}H NMR spectrum of compound **5**
Fig. S 12: ^{1}H NMR spectrum of compound **6**
Fig. S 13: ^{1}H NMR spectrum of compound **7**
Fig. S 14: ^{1}H NMR spectrum of compound **8**
Fig. S 15: ^{1}H NMR spectrum of compound **9**
Fig. S 16: ^{1}H NMR spectrum of compound **10**
Fig. S 17: ^{1}H NMR spectrum of compound **11**
Fig. S 18: ^{1}H NMR spectrum of compound **12**
Fig. S 19: ^{1}H NMR spectrum of compound **13**
Fig. S 20: ^{1}H NMR spectrum of compound **14**
Fig. S 21: ^{1}H NMR spectrum of compound **15**
Fig. S 22: ^{1}H NMR spectrum of compound **16**
Fig. S 23: Graph of IC_{50} determination for compound **1**, inhibition of AChE.
Fig. S 24: Graph of IC_{50} determination for compound **2**, inhibition of AChE.
Fig. S 25: Graph of IC_{50} determination for compound **3**, inhibition of AChE.
Fig. S 26: Graph of IC_{50} determination for compound **11**, inhibition of BuChE.
Fig. S 27: Graph of IC_{50} determination for compound **12,** inhibition of BuChE.
Fig. S 28: Graph of IC_{50} determination for compound **16**, inhibition of AChE.
Fig. S 29: Graph of IC_{50} determination for **galantamine**, inhibition of AChE.
Fig. S 30: Graph of IC_{50} determination for **galantamin**e, inhibition of BuChE.

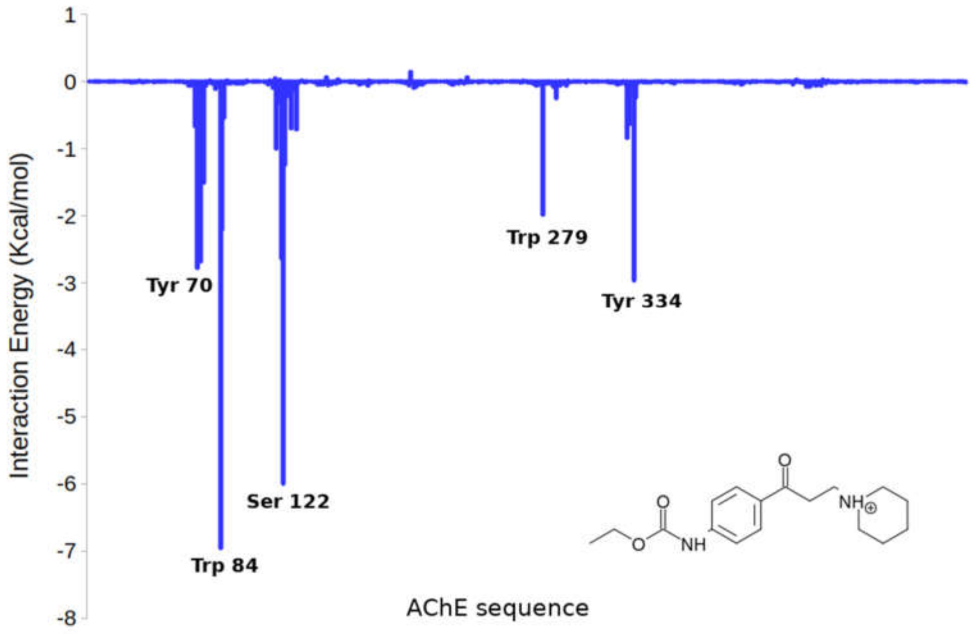

*Fig. S 1: Histogram of interaction energies partitioned with respect to the AChE amino acids in complex with compound **2.** The x-axis denotes the residue number of AChE, and the y-axis denotes the interaction energy between the compounds and specific residue. The negative values and positive values are favorable or unfavorable to binding, respectively.*

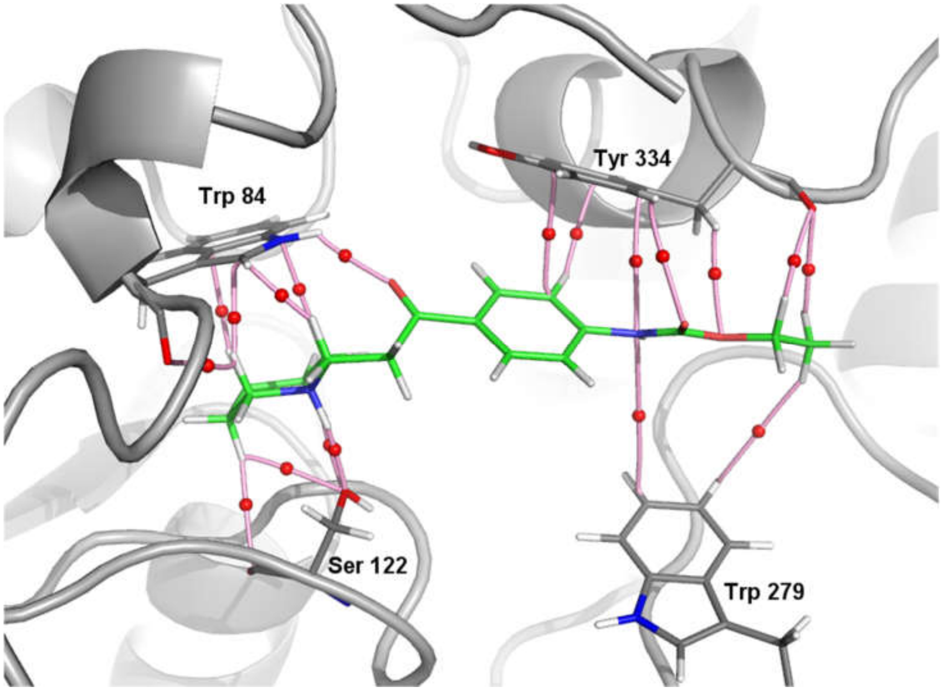

*Fig. S 2: Molecular graph of the non-covalent interactions between the main residues of AChE with compound **2** (green sticks). The elements of the electron density topology are shown. The bond paths connecting the nuclei are represented in pink sticks and the bond critical points are shown as red spheres.*

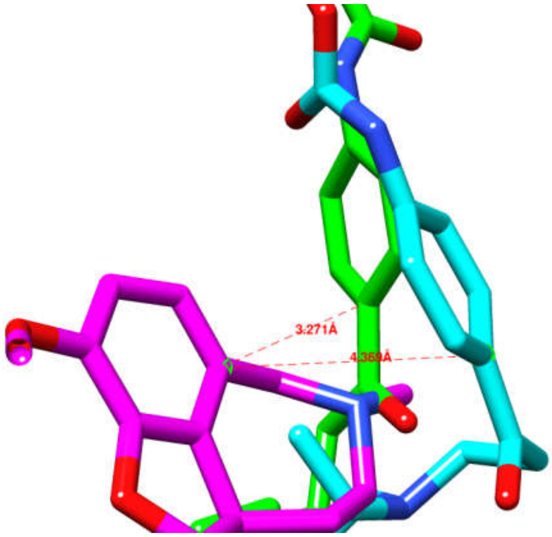

*Fig. S 3: The distances [Å] for the center of mass of molecules **3** and **6** with respect to galantamine. The detail of the spatial view of the compounds bonded in the binding pocket of AChE (PDB code: 1DX6). Compounds **3** (green) and **6** (light blue) are superimposed on galantamine (magenta). The poses of compounds shown in Figure S3 were obtained from the MD simulations.*

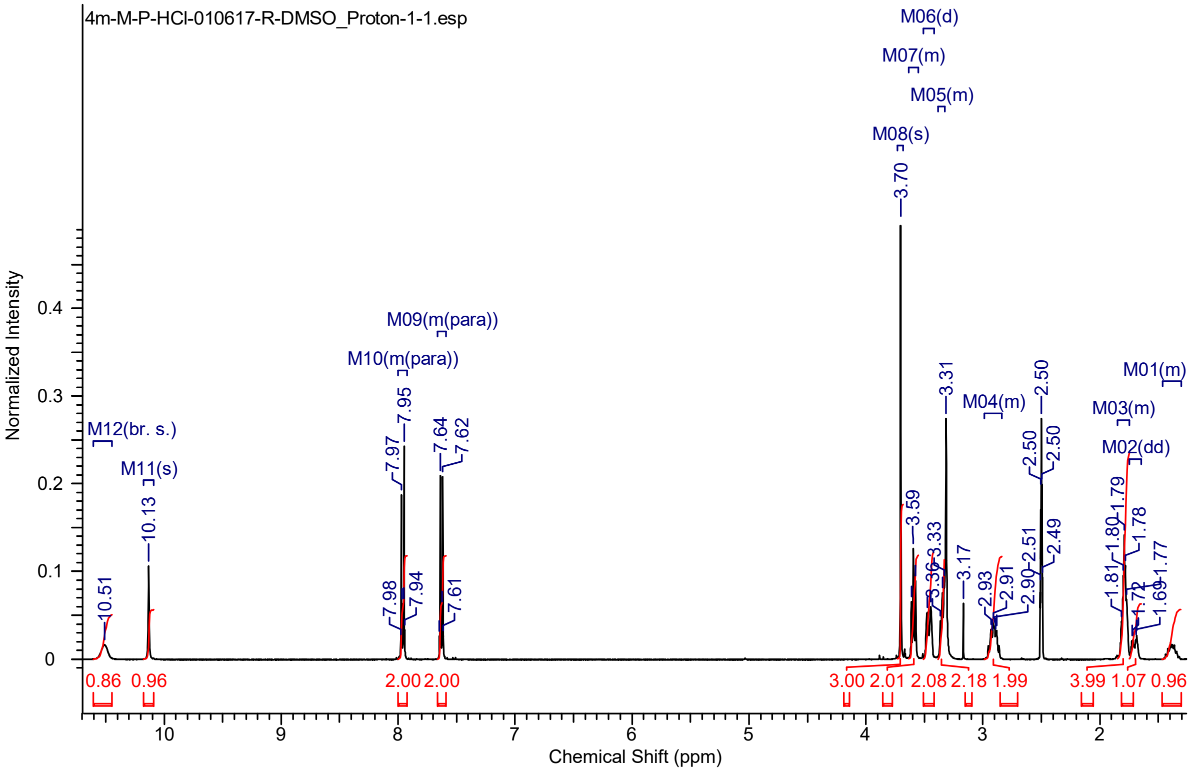

Fig. S 4: ^{1}H NMR spectrum of compound ***1***

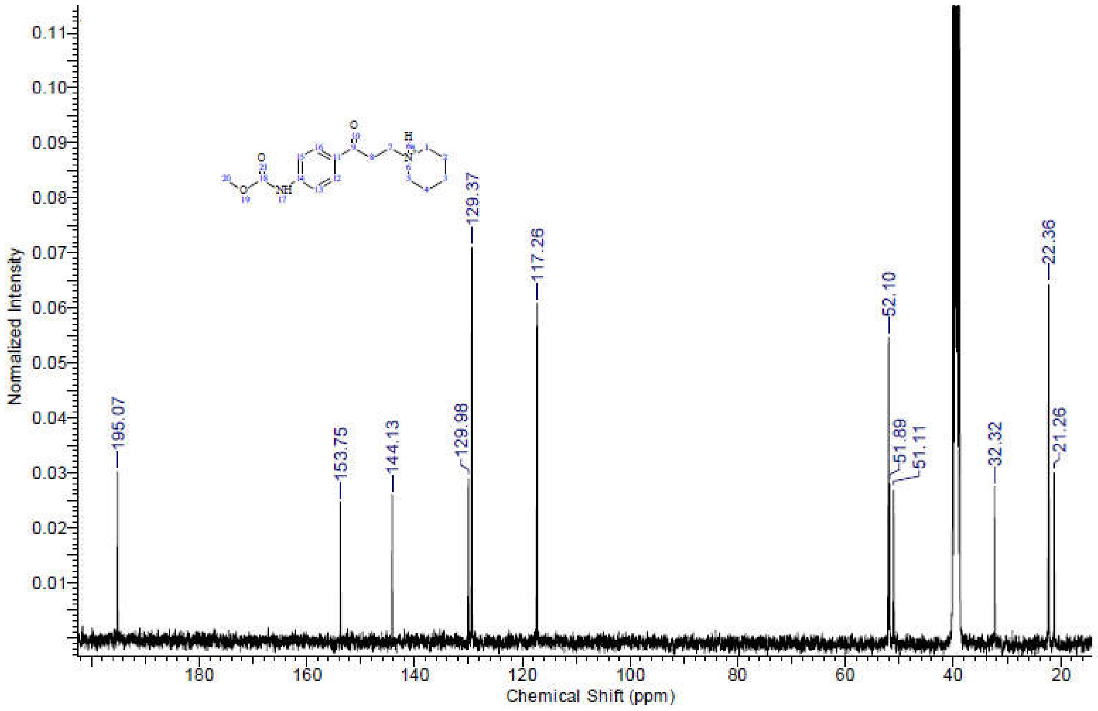

Fig. S 5: ^{13}C NMR spectrum of compound ***1***

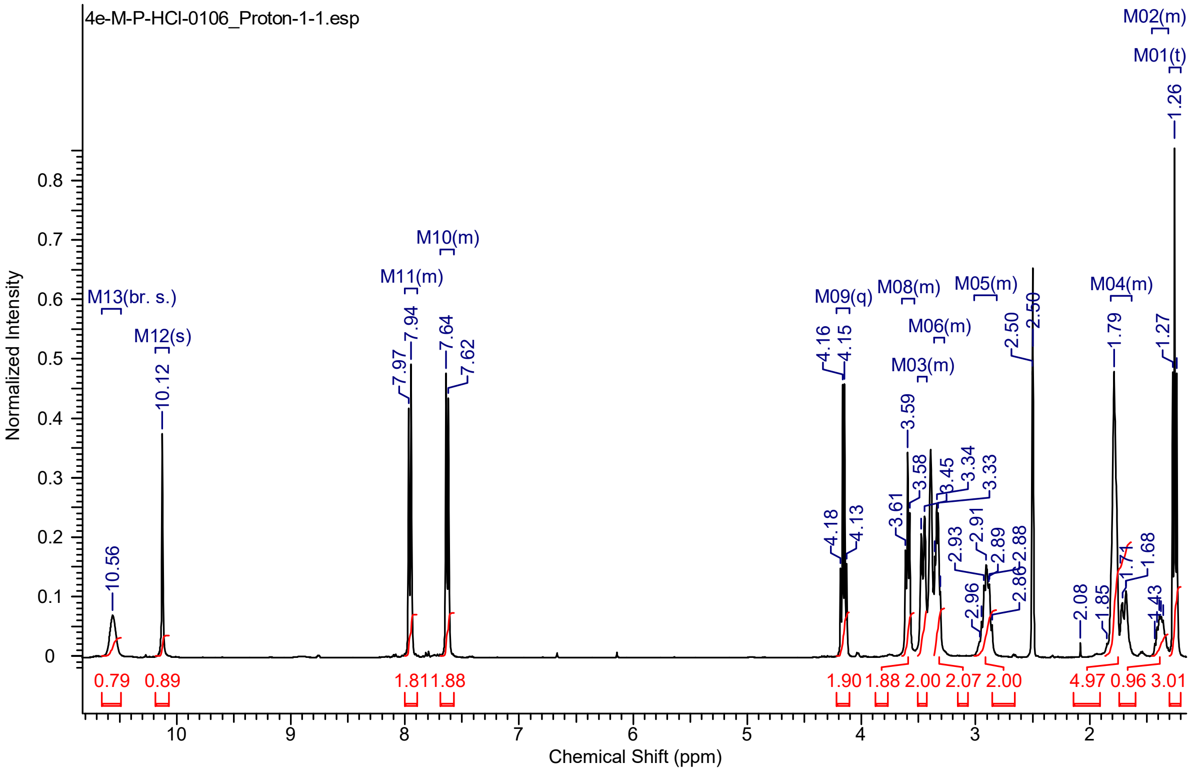

*Fig. S 6: ^{1}H NMR spectrum of compound **2***

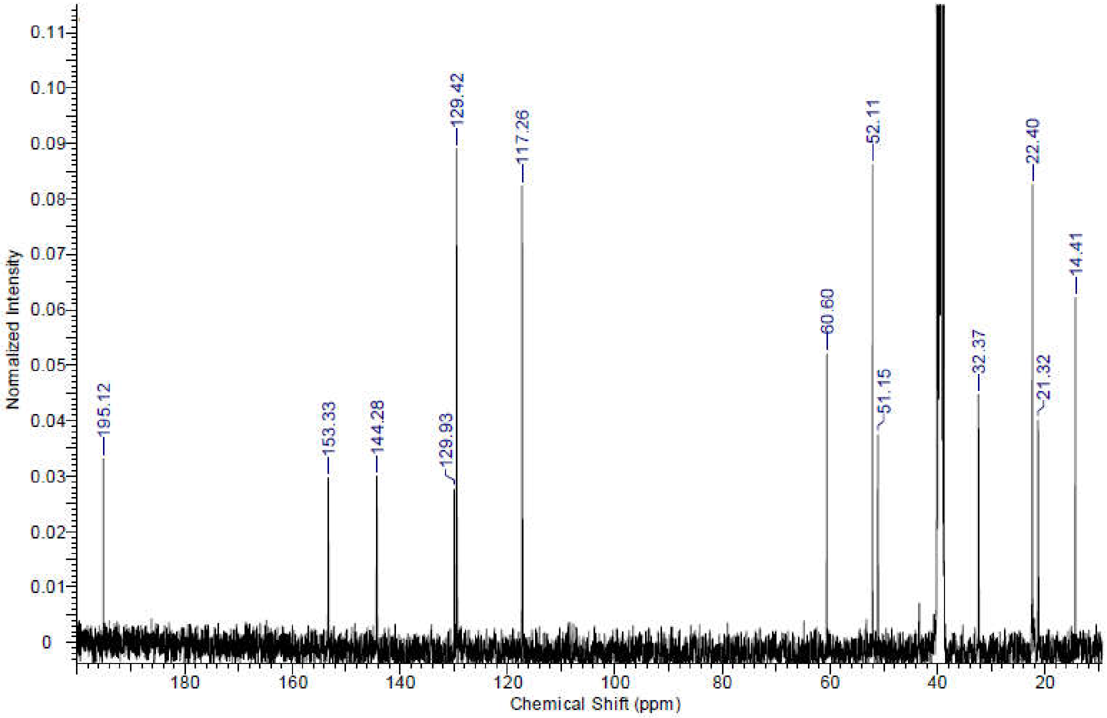

*Fig. S 7: ^{13}C NMR spectrum of compound **2***

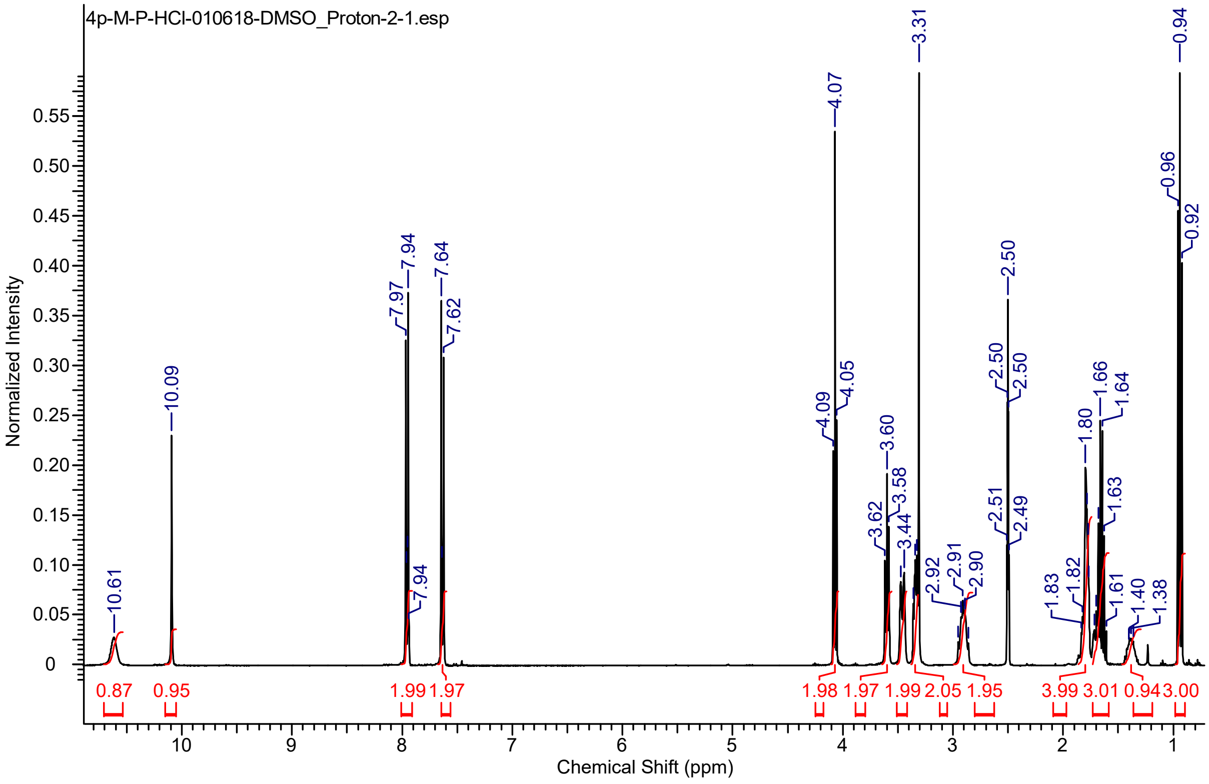

*Fig. S 8: ¹H NMR spectrum of compound **3***

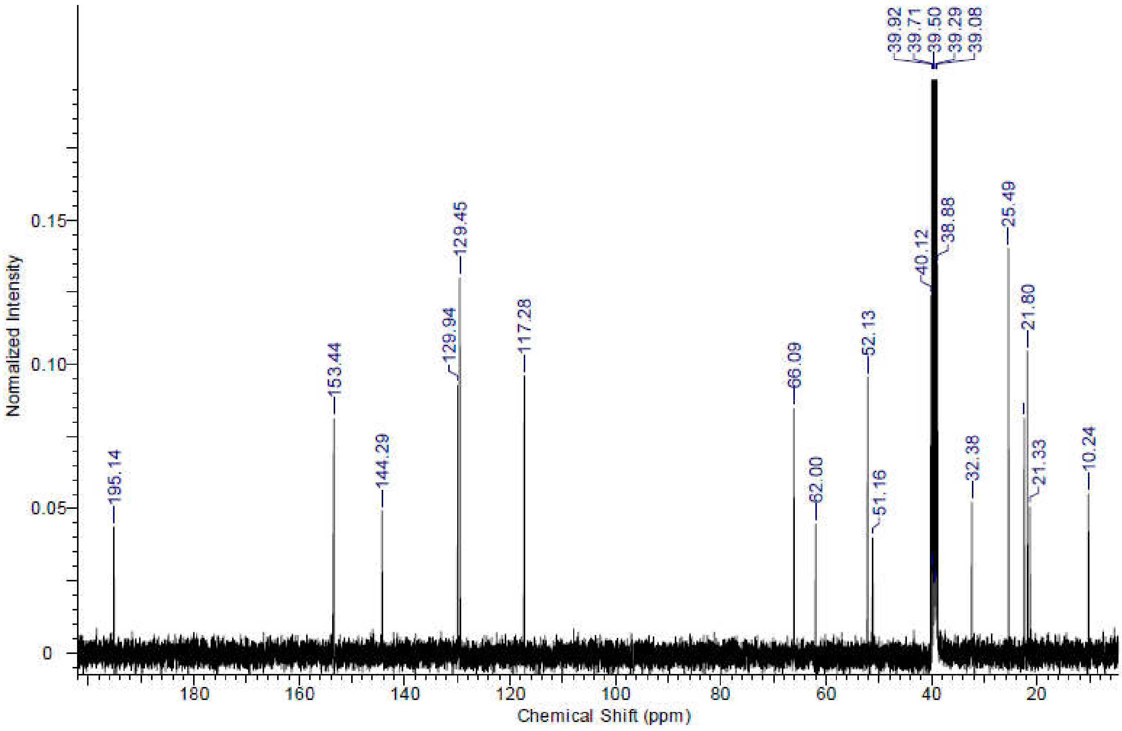

*Fig. S 9: ¹³C NMR spectrum of compound **3***

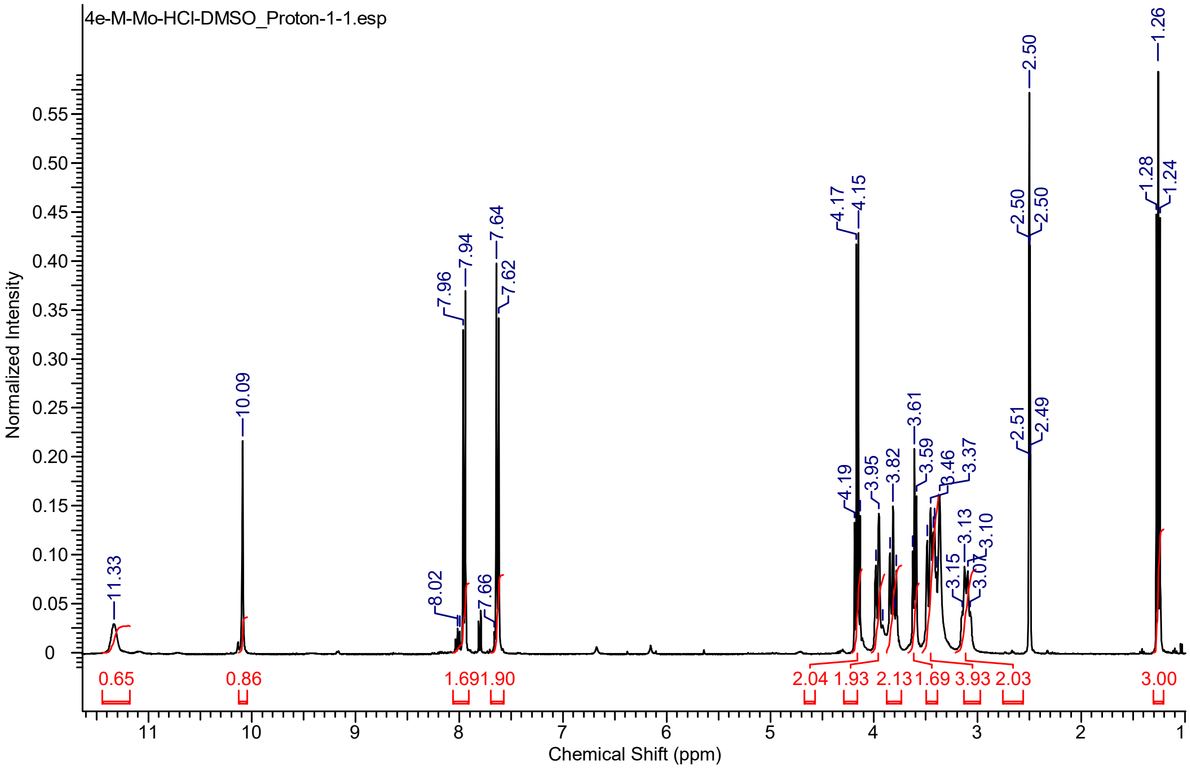

*Fig. S 10: ^{1}H NMR spectrum of compound **4***

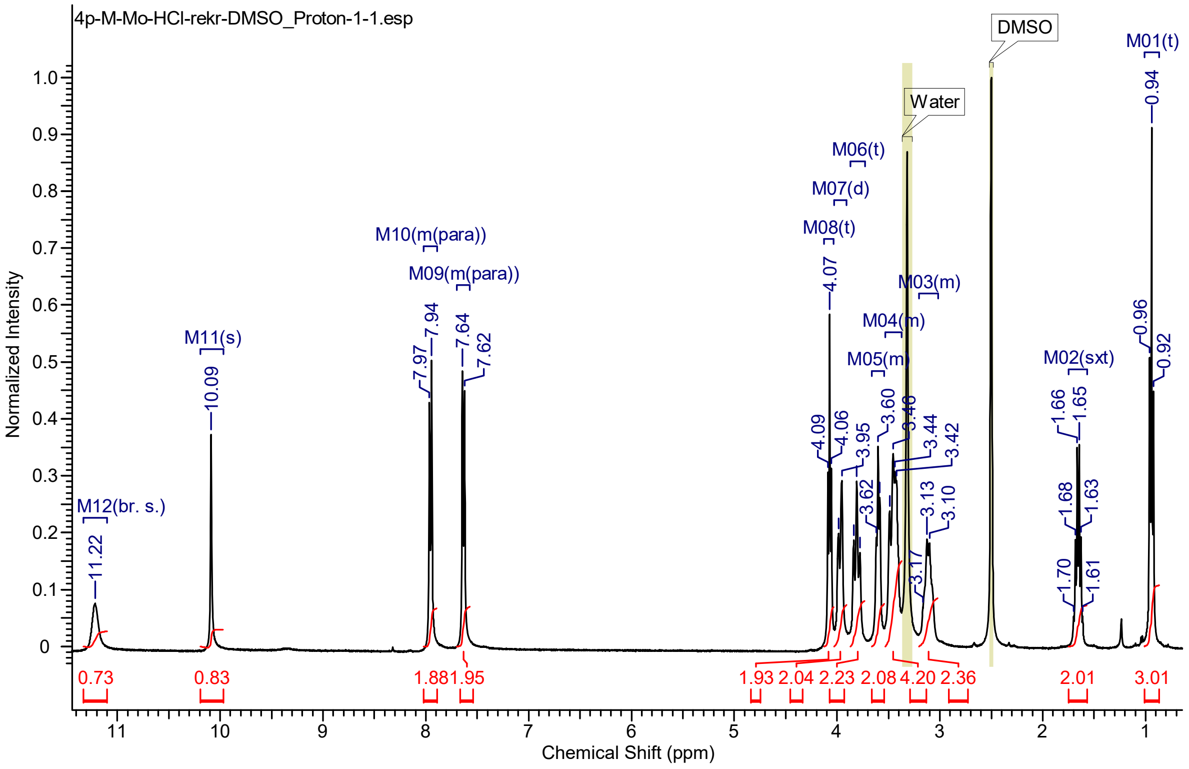

*Fig. S 11: ^{1}H NMR spectrum of compound **5***

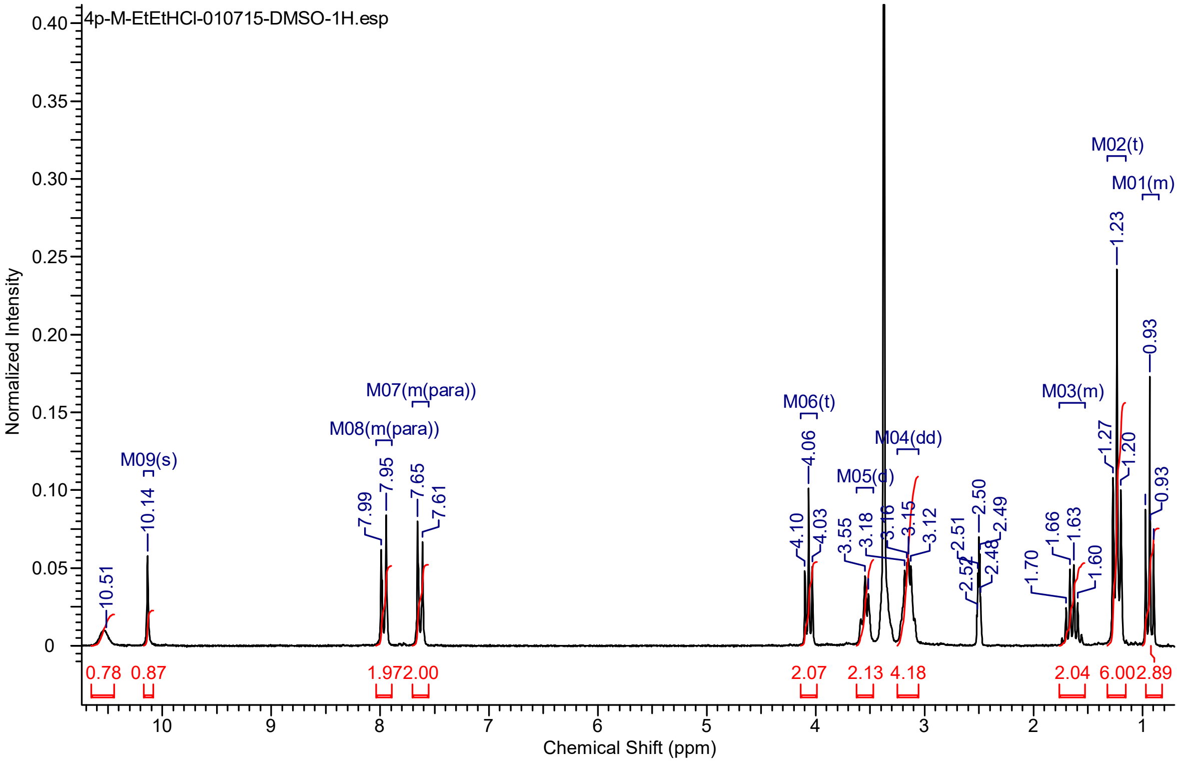

Fig. S 12: ^{1}H NMR spectrum of compound **6**

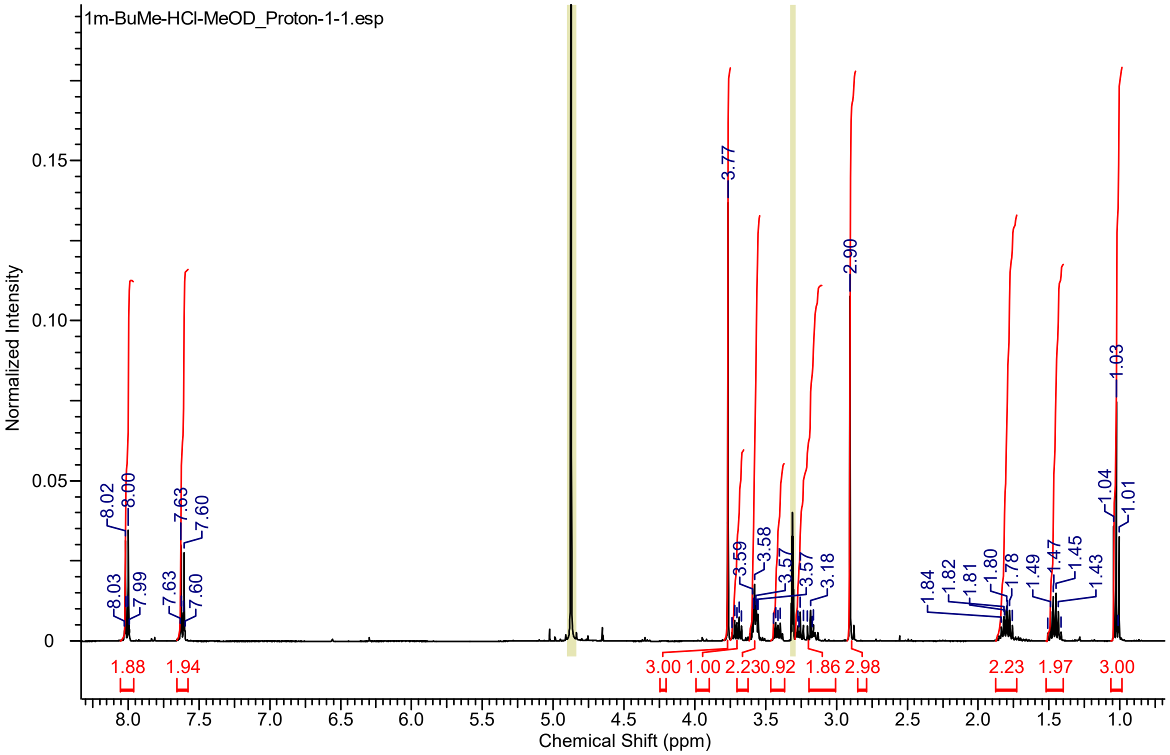

Fig. S 13: ^{1}H NMR spectrum of compound **7**

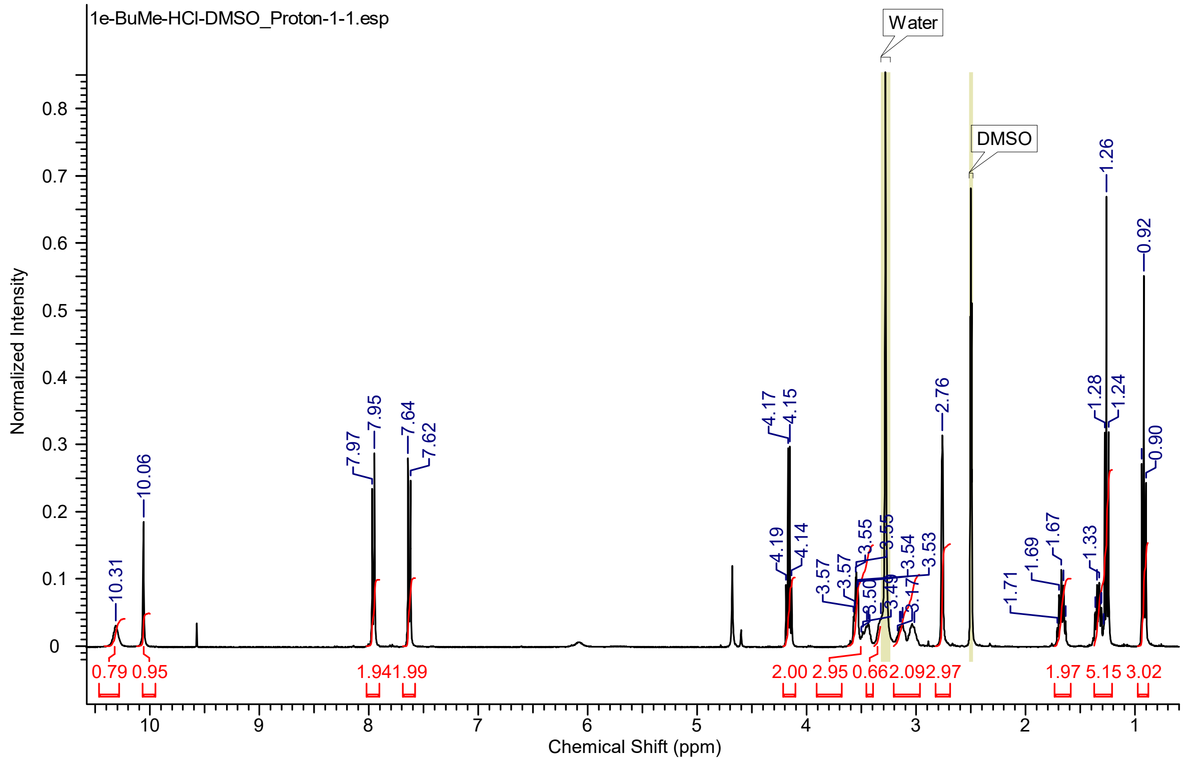

*Fig. S 14: ^{1}H NMR spectrum of compound **8***

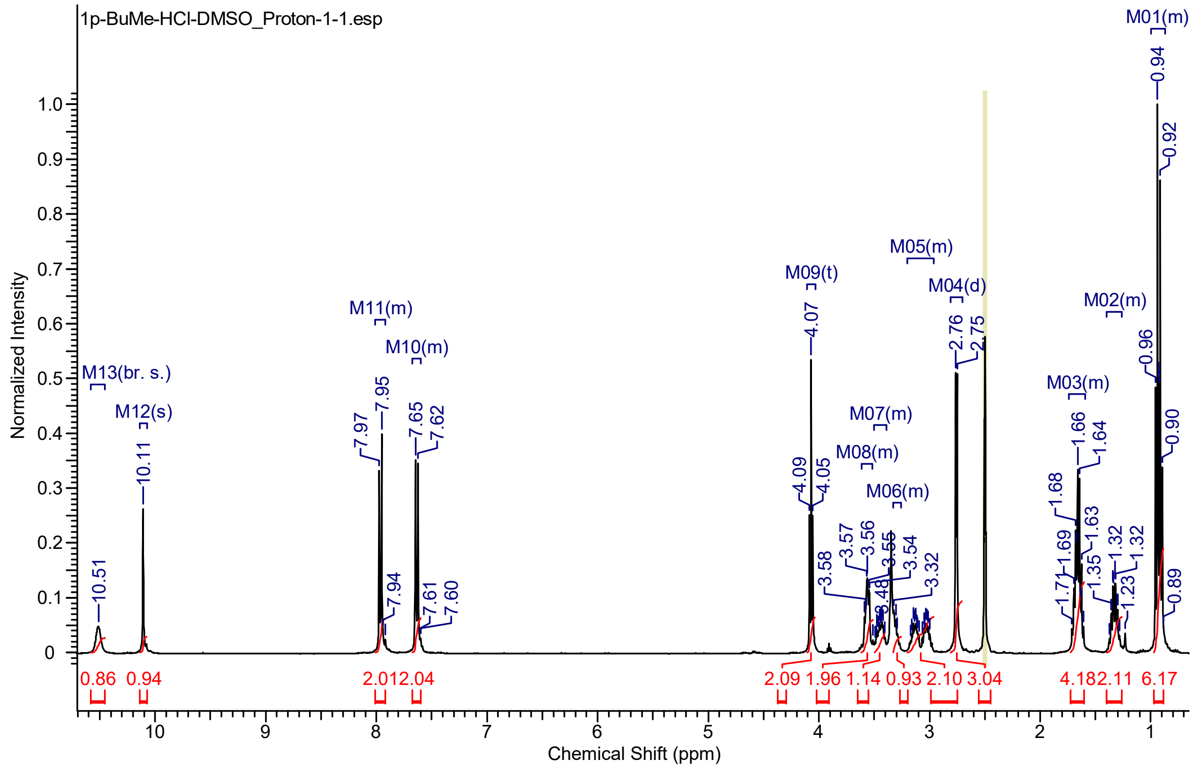

*Fig. S 15: ^{1}H NMR spectrum of compound **9***

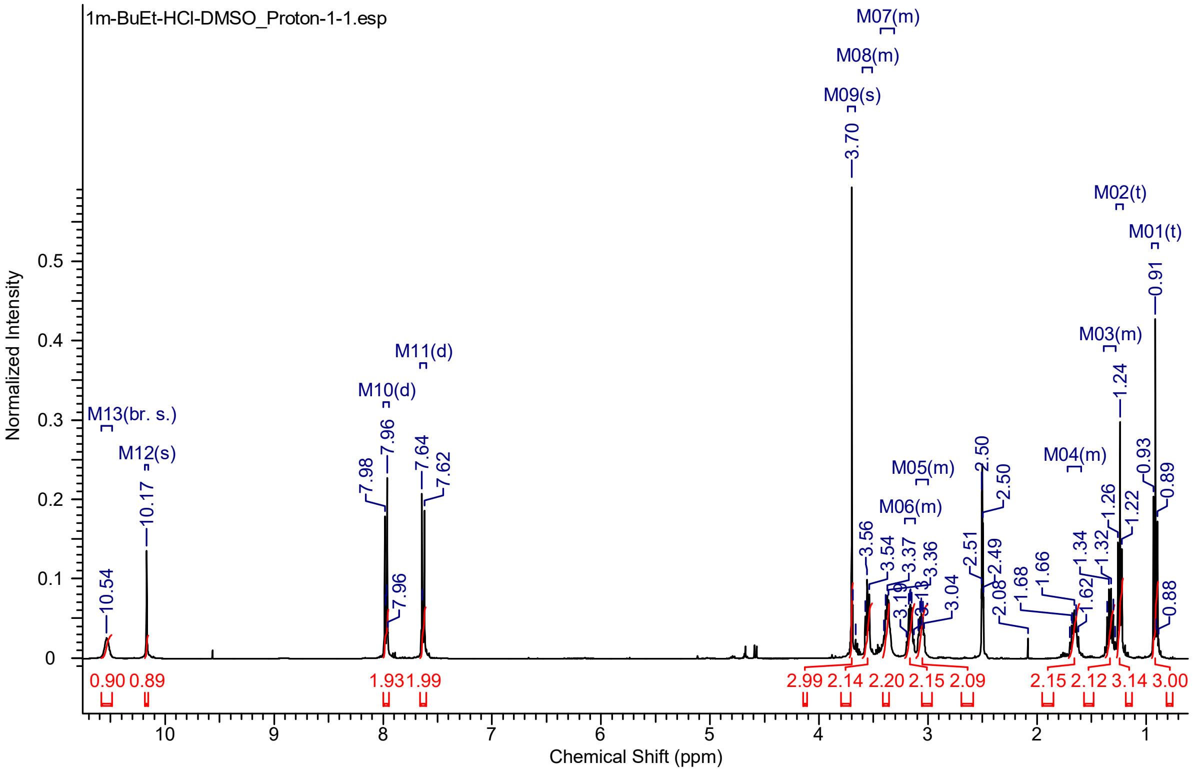

*Fig. S 16: ¹H NMR spectrum of compound **10***

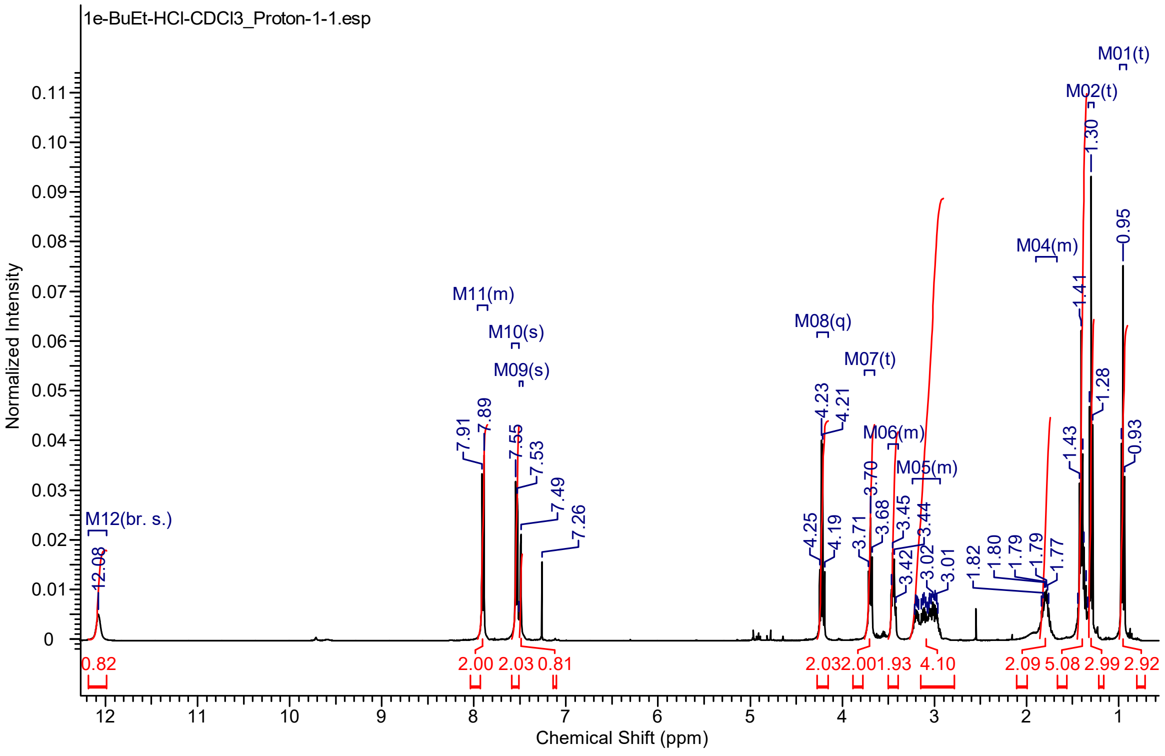

*Fig. S 17: ¹H NMR spectrum of compound **11***

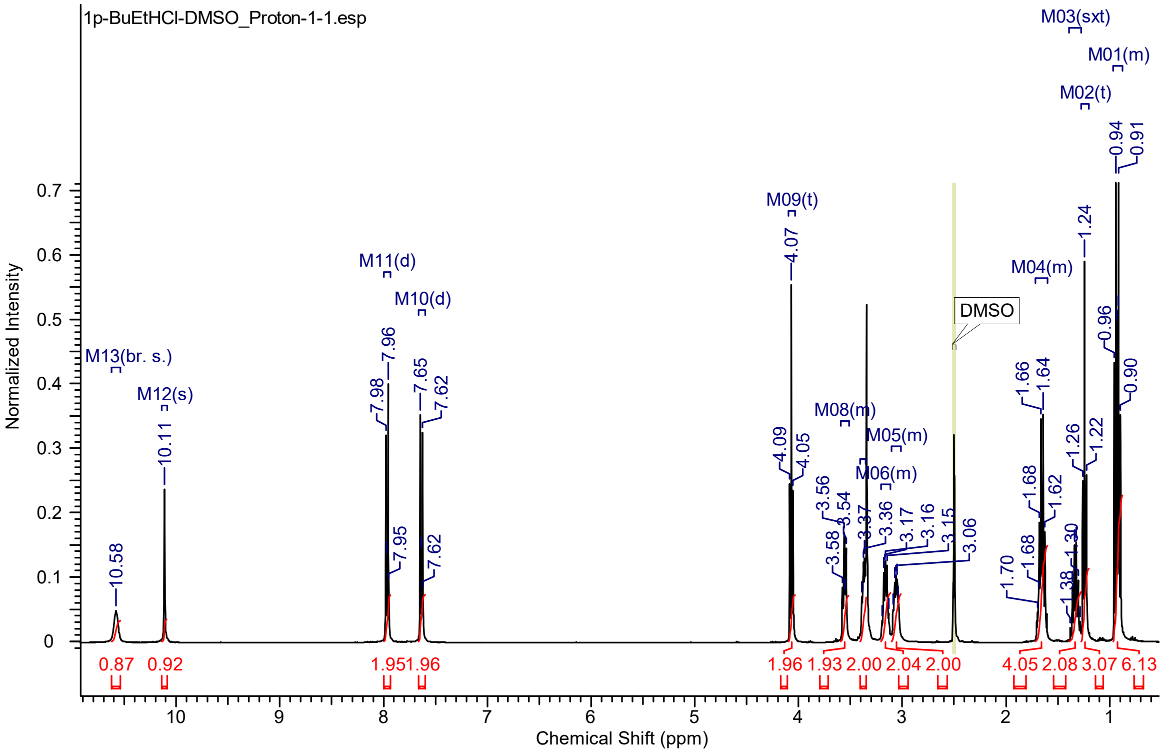

*Fig. S 18: ^{1}H NMR spectrum of compound **12***

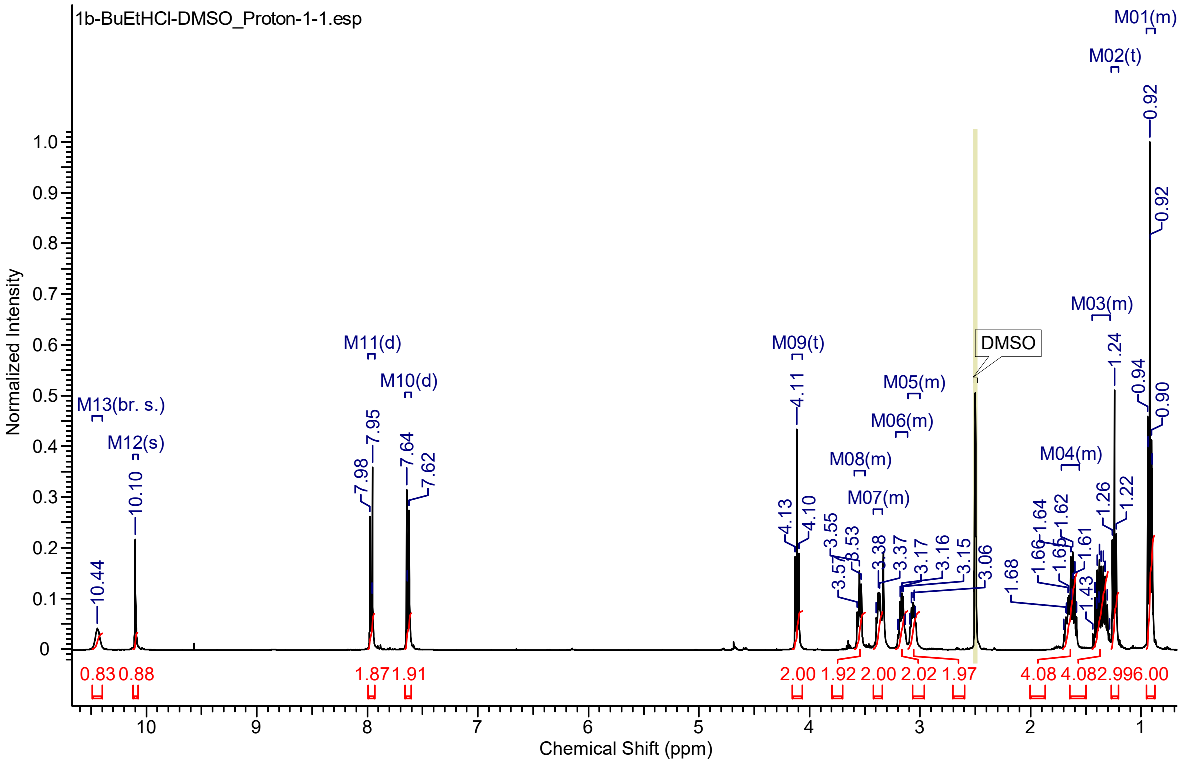

*Fig. S 19: ^{1}H NMR spectrum of compound **13***

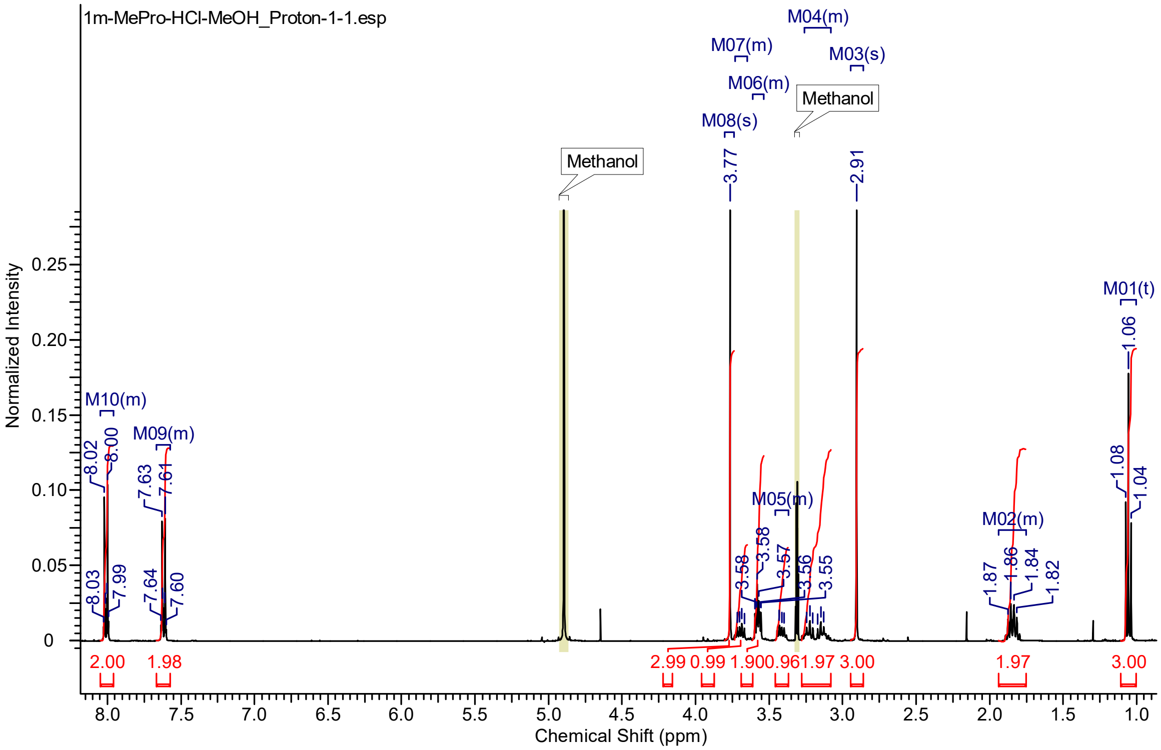

*Fig. S 20: ¹H NMR spectrum of compound **14***

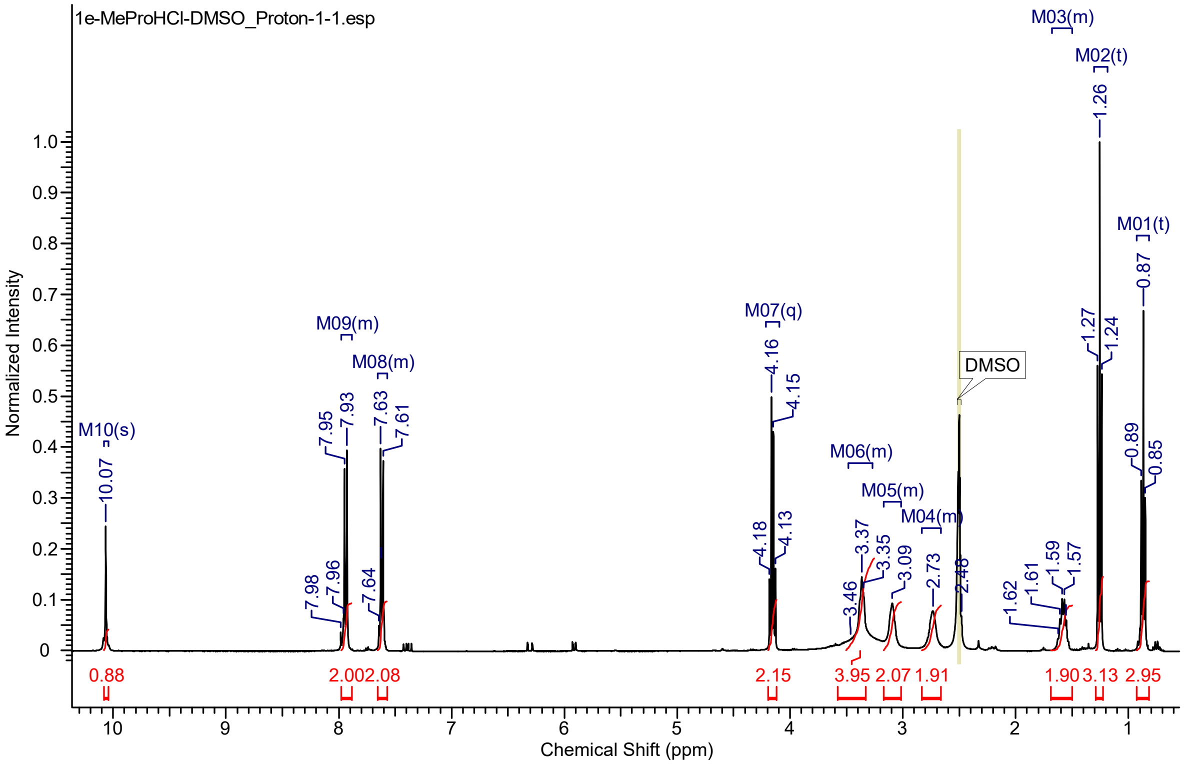

*Fig. S 21: ¹H NMR spectrum of compound **15***

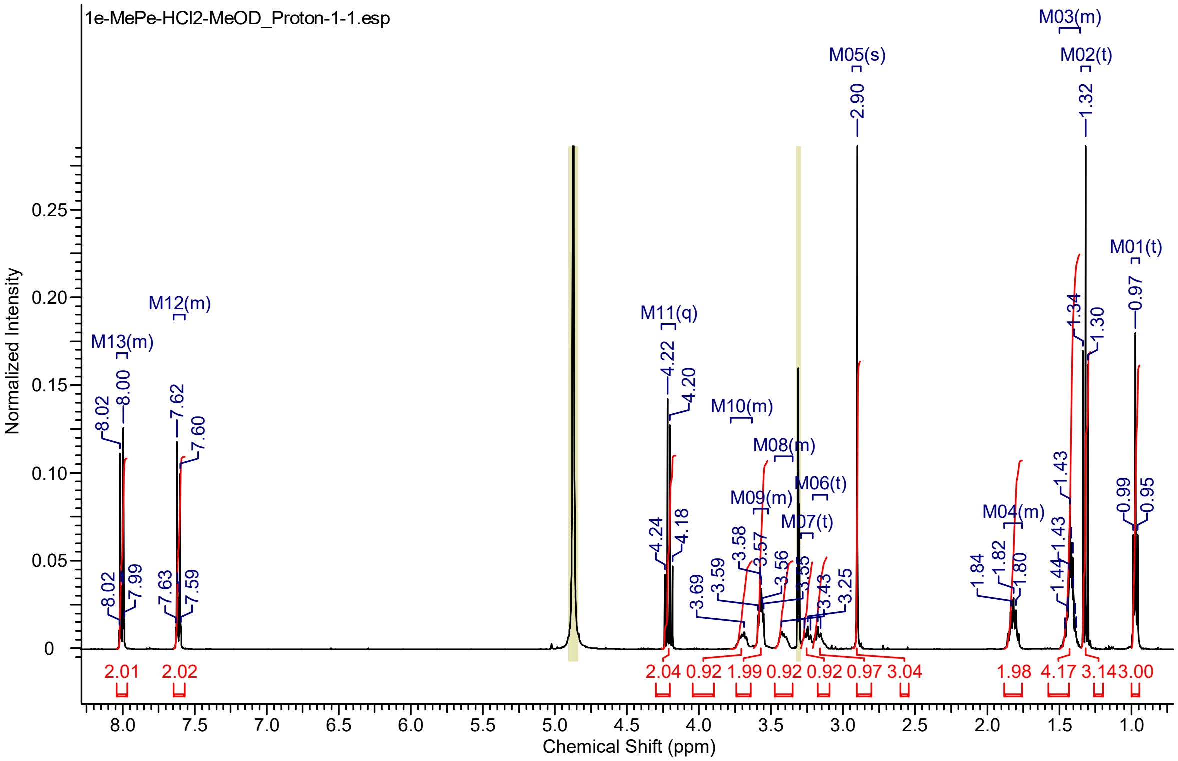

Fig. S 22: ¹H NMR spectrum of compound ***16***

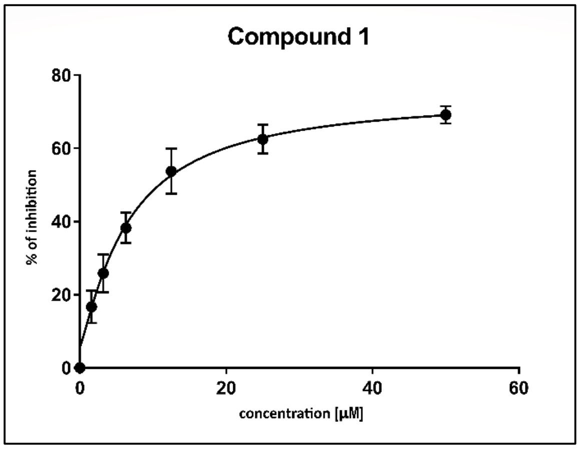

Fig. S 23: Graph of IC_{50} determination for compound ***1****, inhibition of AChE.*

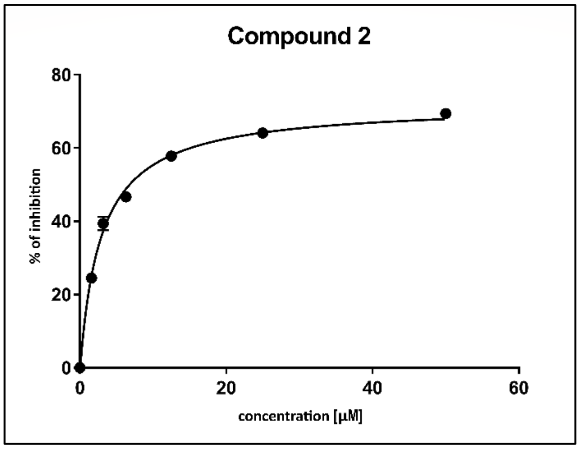

*Fig. S 24: Graph of IC_{50} determination for compound **2**, inhibition of AChE.*

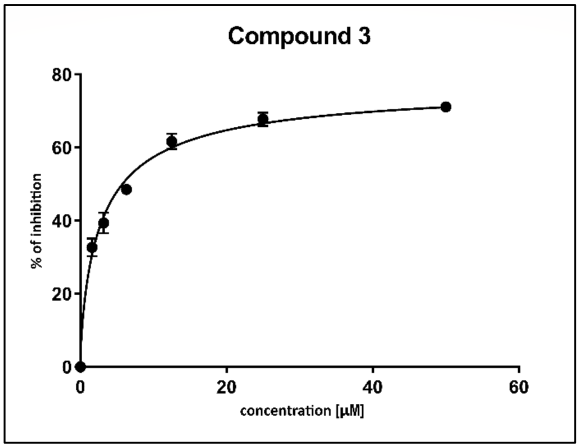

*Fig. S 25: Graph of IC_{50} determination for compound **3**, inhibition of AChE.*

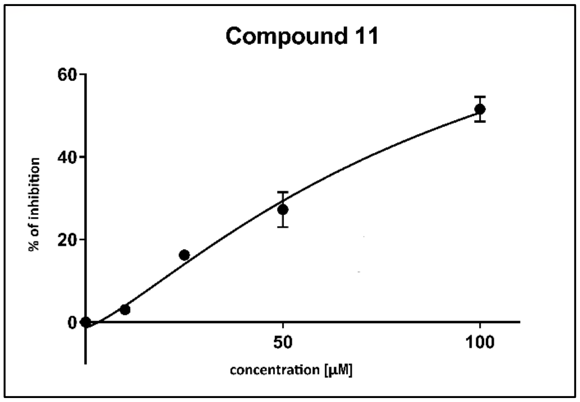

*Fig. S 26: Graph of IC_{50} determination for compound **11**, inhibition of BuChE.*

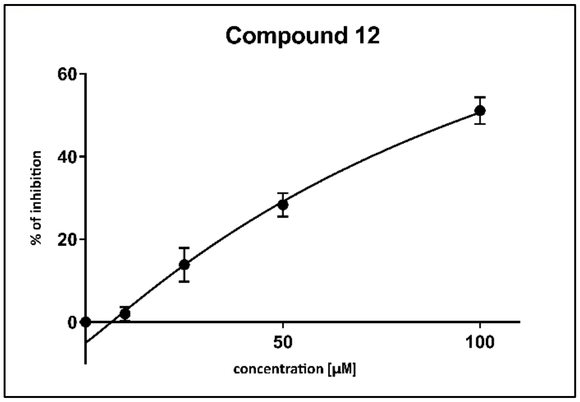

Fig. S 27: Graph of IC_{50} determination for compound ***12,*** *inhibition of BuChE.*

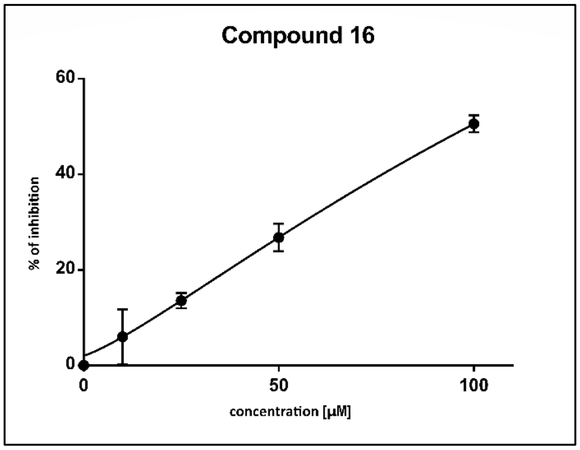

Fig. S 28: Graph of IC_{50} determination for compound ***16****, inhibition of AChE.*

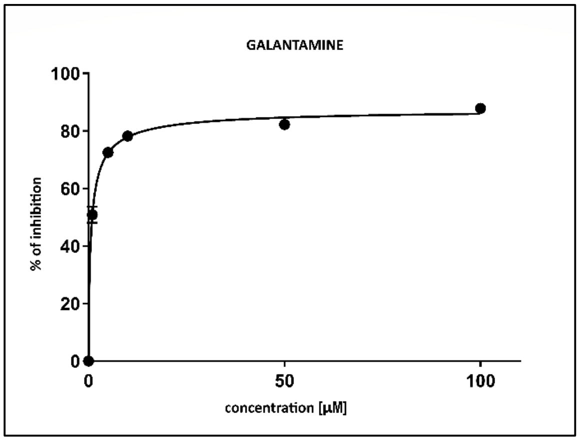

Fig. S 29: Graph of IC_{50} determination for ***galantamine****, inhibition of AChE.*

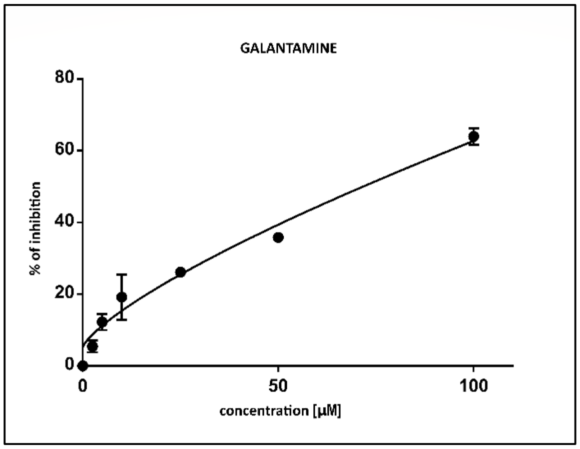

*Fig. S 30: Graph of IC_{50} determination for **galantamine**, inhibition of BuChE.*
